# Evolutionary Divergence of an Ethylene‐Responsive Transcriptional Cascade Governs a Dose‐Dependent Balance between Cotton Fiber Length and Strength

**DOI:** 10.1002/advs.202514154

**Published:** 2025-11-07

**Authors:** Jie Zhang, Mengli Yu, Yuling Guo, Jie Li, Gailing Gu, Ghulam Hussain, Shuya Ma, Fuguang Li, Hongwei Xue, Zhaoen Yang, Zuoren Yang

**Affiliations:** ^1^ State Key Laboratory of Cotton Bio‐breeding and Integrated Utilization Institute of Cotton Research Chinese Academy of Agricultural Sciences Anyang 455000 China; ^2^ Zhengzhou Research Base State Key Laboratory of Cotton Bio‐breeding and Integrated Utilization School of Agricultural Sciences Zhengzhou University Zhengzhou 450001 China; ^3^ Engineering Research Centre of Cotton Ministry of Education/College of Agriculture Xinjiang Agricultural University 311 Nongda East Road Urumqi 830052 China; ^4^ Shanghai Collaborative Innovation Center of Agri‐Seeds/Joint Center for Single Cell Biology School of Agriculture and Biology Shanghai Jiao Tong University Shanghai 200240 China; ^5^ Xinjiang Key Laboratory of Crop Gene Editing and Germplasm Innovation Institute of Western Agricultural of CAAS Changji Xinjiang 831100 China

**Keywords:** 1‐aminocyclopropane‐1‐carboxylic synthase (ACS), cotton, ethylene, fiber elongation, secondary cell wall (SCW), transcriptional regulation

## Abstract

Ethylene is a key hormone in plant development, but how its endogenous levels quantitatively regulate the dose‐dependent balance between cotton fiber length and strength has been poorly understood. Here, it is shown that natural variations in ethylene content across *Gossypium* species (*G. hirsutum*, *G. arboreum*, and *G. raimondii*) correlate with their distinct fiber length and secondary cell wall (SCW) attributes. A post‐translational mechanism is identified where a kinase‐deficient variant of CASEIN KINASE1 (PK1) stabilizes key ACS1 isoforms to enhance ethylene biosynthesis. In tetraploid cotton (*G. hirsutum*), elevated ethylene inhibits elongation but promotes SCW deposition, yielding shorter, stronger fibers, while suppressing *GhACS1* impairs both processes. Mechanistically, a hierarchical GhEIN3‐GhERF‐GhCOBL4 transcriptional cascade is uncovered that orchestrates this balance. Remarkably, elevating ethylene in the diploid ancestor *G. arboreum* elicits the opposite phenotype: longer, thinner fibers. This is explained by a functional inversion in the transcriptional response of the physically conserved cascade, which is activated in *G. hirsutum* but repressed in *G. arboreum*. The findings establish a tunable module governing a dose‐dependent balance between fiber length and strength, and its evolutionary divergence provides novel targets to break this developmental constraint in cotton engineering.

## Graphical abstract

A tunable, ethylene‐responsive cascade governing the dose‐dependent balance between fiber length and strength is unveiled. Elevated ethylene strengthens modern tetraploid cotton (*G. hirsutum*) fibers but elongates those of its diploid ancestor (*G. arboreum*). This phenotypic switch is traced to an evolutionary functional inversion of a conserved EIN3‐ERF‐COBL4 module, offering novel strategies to break this developmental constraint.

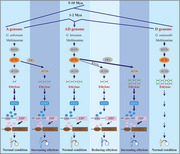

## 1 Introduction

Cotton (*Gossypium* spp.) is the world's most important natural fiber crop, and improving fiber quality without compromising yield remains a central goal of modern breeding.^[^ ^1^ ^]^ Upland cotton (*G. hirsutum*), an allotetraploid species, dominates global production. It originated from the natural hybridization and subsequent polyploidization between an A‐genome diploid, represented by *G. arboreum*, and a D‐genome diploid, with *G. raimondii* as its extant progenitor. While *G. hirsutum* is prized for its high lint yield and adaptable fiber properties, its diploid ancestors harbor distinct and valuable fiber‐related alleles. *G. arboreum* (A‐genome) typically produces shorter and coarser fibers but offers genetic resources for unique fiber attributes and superior stress tolerance.^[^ ^2^, ^3^ ^]^ Conversely, *G. raimondii* (D‐genome), though not producing spinnable lint itself, is a critical source of genes conferring enhanced fiber fineness, which have contributed to the qualities of modern *G. hirsutum*.^[^ ^4^ ^]^ Harnessing this rich natural variation from both the cultivated tetraploid and its ancestral diploids requires a detailed understanding of the genetic and hormonal networks that govern fiber development.

Fiber morphogenesis proceeds through two sequential but tightly coupled phases: rapid cell elongation followed by massive secondary cell wall (SCW) cellulose deposition.^[^ ^5^ ^]^ Ethylene is a key hormonal regulator of both stages. Classical pharmacological and transgenic studies showed that exogenous ethylene or its precursor 1‐aminocyclopropane‐1‐carboxylic acid (ACC) can promote fiber elongation, whereas inhibitors shorten fibers.^[^ ^6^, ^7^ ^]^ Ethylene also stimulates H_2_O_2_ production and activates transcription factors such as WLIM1a or GhERF108, thereby triggering SCW biosynthesis.^[^ ^8^, ^9^ ^]^ Nevertheless, the relationship between endogenous ethylene levels and fiber traits is far from linear: both deficient and excessive ethylene can restrict elongation or alter wall architecture.^[^ ^10^ ^]^ The molecular basis of this biphasic, dosage‐dependent response is largely unknown.

Ethylene biosynthesis is controlled at the first committed step, the conversion of S‐adenosyl methionine (SAM) to ACC by ACC synthase (ACS).^[^ ^11^, ^12^ ^]^ ACS proteins are encoded by a multigene family and subdivided into three types that differ in their C‐terminal regulatory motifs.^[^ ^13^ ^]^ Their abundance is controlled transcriptionally, for example, by SlRIN in tomato or WRKY33 in Arabidopsis,^[^ ^14^, ^15^ ^]^ and also post‐translationally. In Arabidopsis, phosphorylation of type‐I ACS stabilizes the enzyme,^[^ ^16^ ^]^ whereas phosphorylation of type‐II ACS5 by CASEIN KINASE 1.8 (CK1.8) promotes its ubiquitin‐mediated degradation.^[^ ^17^ ^]^ Ubiquitination of type‐III ACS7 by XBAT32 likewise limits ethylene output.^[^ ^18^ ^]^ Whether a similar post‐translational mechanism modulates cotton‐specific ACS isoforms during fiber development has not been examined.

Ethylene perception and signal transduction are mediated by endoplasmic reticulum‐localized ethylene receptors (ETRs) that repress downstream signaling via the Raf‐like kinase constitutive triple response 1 (CTR1). Ethylene binding inactivates CTR1, releases the membrane protein ethylene insensitive 2 (EIN2), and ultimately stabilizes ethylene insensitive 3/ethylene insensitive 3‐like (EIN3/EIL) transcription factors, which activate ethylene response factors (ERFs) and other target genes.^[^ ^19^, ^20^ ^]^ Several ERFs have been linked to cotton fiber growth: GhERF108 promotes SCW deposition through interaction with ARF7,^[^ ^9^ ^]^ whereas GhERF41 orchestrates both primary cell wall (PCW) and SCW biosynthesis by activating PCW‐ and SCW‐associated *CESA* genes to enhance fiber length and strength.^[^ ^21^ ^]^ Despite these advances, the transcriptional hierarchy that connects ethylene signaling to the fiber development machinery remains poorly understood.

Foundational work by Shi et al^[^ ^22^ ^]^ and Li et al^[^ ^23^ ^]^ established ethylene as a vital positive regulator with dose‐dependent effects on cotton fiber development. However, these pioneering studies often lacked experimental validation derived from direct manipulation of in vivo ethylene levels within the fibers, and did not account for the molecular intricacies underlying ethylene's dosage‐dependent influence, particularly in mediating the crucial balance between fiber elongation and the subsequent transition to SCW deposition. Consequently, how cotton fibers precisely orchestrate this balance, which ultimately dictates fiber length and strength, remains a pivotal unanswered question.

Here, we aim to resolve how endogenous ethylene dosage quantitatively coordinates the balance between elongation and SCW deposition in cotton fibers, and whether this control logic has diverged across *Gossypium* lineages. By profiling three cotton species with contrasting fiber phenotypes, we first establish a correlation between natural variation in endogenous ethylene content and species‐specific differences in fiber length and SCW thickness. To manipulate ethylene dosage in vivo with temporal and tissue specificity while preserving canonical signal transduction, we leveraged a previously characterized kinase‐deficient Arabidopsis CK1.8^D128N^ variant,^[^ ^17^ ^]^ expressed specifically in cotton fibers (hereafter PK1). We show that heterologous PK1 engages the fiber‐dominant GhACS1 isoforms to elevate ethylene production. Downstream, we define a hierarchical transcriptional cascade where GhEIN3‐D08 directly activates *GhERF‐A11*, which in turn targets *GhCOBL4‐A08* to coordinate this balance. Furthermore, we assess conservation versus divergence of this ethylene‐responsive module between the tetraploid (*G. hirsutum*) and its diploid ancestor (*G. arboreum*) to evaluate how evolutionary rewiring might underlie contrasting fiber phenotypes. Collectively, our findings establish a tunable, ethylene‐responsive module that governs the dose‐dependent balance between fiber length and strength and, through its evolutionary divergence, provide novel strategies and molecular targets to decouple these traits in cotton breeding.

## 2 Results

### 2.1 Ethylene Content is Associated with Fiber Characteristics in Three Cotton Species

**Figure 1:**
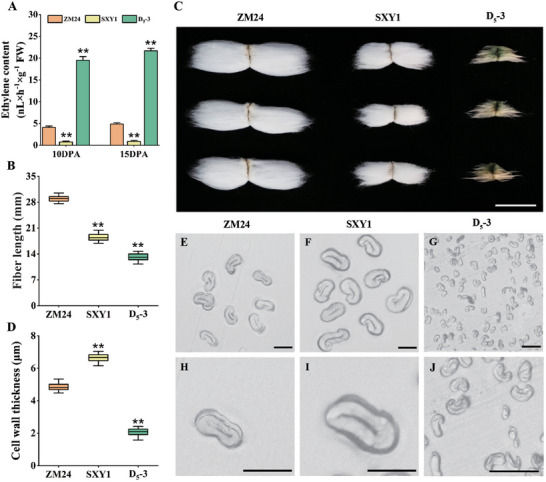
Comparison of ethylene content, fiber length, and cell wall thickness for *Gossypium hirsutum*, *Gossypium arboreum*, and *Gossypium raimondii*. A) The ethylene content in 10 and 15 DPA fibers of three cotton species was measured, with at least 30 fibers measured per replicate. B) Measurement and statistical analysis of mature fiber length from three cotton species (n > 30). C) Representative images of mature fibers from three cotton species. Bar = 2 cm. D) Cell wall thickness of mature fibers from three cotton species (n > 30). E–G) Cross‐sectional images of mature fibers from three cotton species. Bar = 10 µm. H–J) Partial magnified images of (E–G). Bar = 10 µm. Mean values and standard deviation of three biological replicates are presented. Independent *t*‐tests demonstrated that there were significant differences (^**^, *P* < 0.01) between *G. hirsutum* and *G. arboreum* as well as between *G. hirsutum* and *G. raimondii* in terms of ethylene content, fiber length, and cell wall thickness.

Previous research from our laboratory revealed significant differences in the expression of ethylene biosynthesis genes among allotetraploid *Gossypium hirsutum* (AD_1_) and its two diploid potential ancestor *Gossypium arboreum* (A_2_) and *Gossypium raimondii* (D_5_).^[^ ^23^ ^]^ To assess the functional impact of these differences, we measured ethylene content in 10 and 15 days post anthesis (DPA) fibers from each species. As shown in Figure **1**A, fibers of *G. arboreum* had significantly lower ethylene levels compared to *G. hirsutum*, while *G. raimondii* fibers showed a marked increase, mirroring patterns of ethylene biosynthesis gene expression.

We further compared fiber traits across the three species. *G. hirsutum* exhibited the longest fibers, whereas *G. arboreum* and *G. raimondii* had significantly shorter fibers, with *G. raimondii* being the shortest (Figure 1B,C). Analysis of SCW thickness revealed that *G. arboreum* had thicker SCWs than *G. hirsutum*, whereas *G. raimondii* had reduced SCW thickness (Figure 1D–J). Viewed together, our findings provide compelling evidence from natural species variation for the hypothesis of ethylene's dose‐dependent effect on fiber development. Both insufficient (in *G. arboreum*) and excessive (in *G. raimondii*) endogenous ethylene levels are correlated with impaired fiber elongation compared to the optimal level in *G. hirsutum*, thus corroborating and extending earlier proposals with robust, evolutionarily relevant data.^[^ ^22^, ^23^ ^]^

### 2.2 Gene Expression Involved in Ethylene Pathway is Highly Divergent across Three Cotton Species

To investigate the molecular basis underlying ethylene content variations among three cotton species at the transcriptional level, we performed transcriptome sequencing on 10 and 15 DPA fibers from *G. hirsutum*, *G. arboreum*, and *G. raimondii*, respectively. Based on the ZM24 genome annotation, we identified genes involved in ethylene biosynthesis and signaling, which were categorized into seven functional groups: *methionine adenosyltransferase/S‐adenosylmethionine* (*MAT/SAM*), *1‐aminocyclopropane‐1‐carboxylate synthase* (*ACS*), *1‐aminocyclopropane‐1‐carboxylic acid oxidase* (*ACO*), *ethylene receptor* (*ETR*), *constitutive triple response* (*CTR*), *ethylene insensitive 2* (*EIN2*), and *ethylene insensitive 3/ethylene insensitive 3‐like* (*EIN3/EIL*). Homologous genes from the two diploid species were then identified through sequence similarity searches (Figure S1, Supporting Information).

Differential expression analysis of the ethylene biosynthesis and signaling genes (|log_2_(fold change)| ≥ 1, Adjusted *P*‐value < 0.05) was performed on 10 and 15 DPA fibers across pairwise species comparisons (*G. hirsutum* vs *G. arboreum* and *G. hirsutum* vs *G. raimondii*), identifying 42 differentially expressed genes (DEGs) in each pairwise comparison. Notably, most DEGs exhibited higher expression in *G. hirsutum* compared to *G. arboreum*, while the opposite trend was observed between *G. hirsutum* and *G. raimondii* (Figure S1 and Tables S2–S5, Supporting Information). qRT‐PCR validation of randomly selected DEGs was consistent with the transcriptome data (Figure S2, Supporting Information). These findings suggest that species‐specific differences in ethylene pathway gene expression likely contribute to ethylene content variation, thereby influencing fiber length and SCW thickness across the three cotton species.

### 2.3 Identification of a Molecular Target to Modulate Ethylene Levels in Upland Cotton Fibers

To validate the hypothesis regarding ethylene's dose‐dependent effect on fiber development, we sought to identify a molecular target capable of modulating endogenous ethylene levels in cotton fibers. Previous studies in Arabidopsis and tomato demonstrated that ethylene biosynthesis can be regulated by manipulating ACS activity through interaction with a kinase‐deficient casein kinase1.8 variant (CK1.8^D128N^), which carries a D128N substitution (aspartic acid to asparagine at position 128).^[^ ^17^ ^]^ Alignment of Arabidopsis *CK1.8^D128N^* with its closest cotton homolog (*GhCK1*) revealed conservation of the kinase domain, with the D128 residue retained as aspartic acid in GhCK1 (Figure S3, Supporting Information). Notably, AtACS5 interacts with CK1.8^D128N^, suggesting that a similar regulatory mechanism may exist in cotton. Comparative analysis of ACS family members revealed eight cotton ACS homologs closely related to AtACS5 (Figure S4, Supporting Information). However, transcriptome data showed that these homologs were either not expressed or expressed at extremely low levels in cotton fibers (Tables S2–S5, Supporting Information). Instead, we identified eight highly expressed *ACS* genes in fibers, belonging to the type I ACS and aminotransferase families, respectively (Figures S4, S5, and Tables S2–S5, Supporting Information).

**Figure 2:**
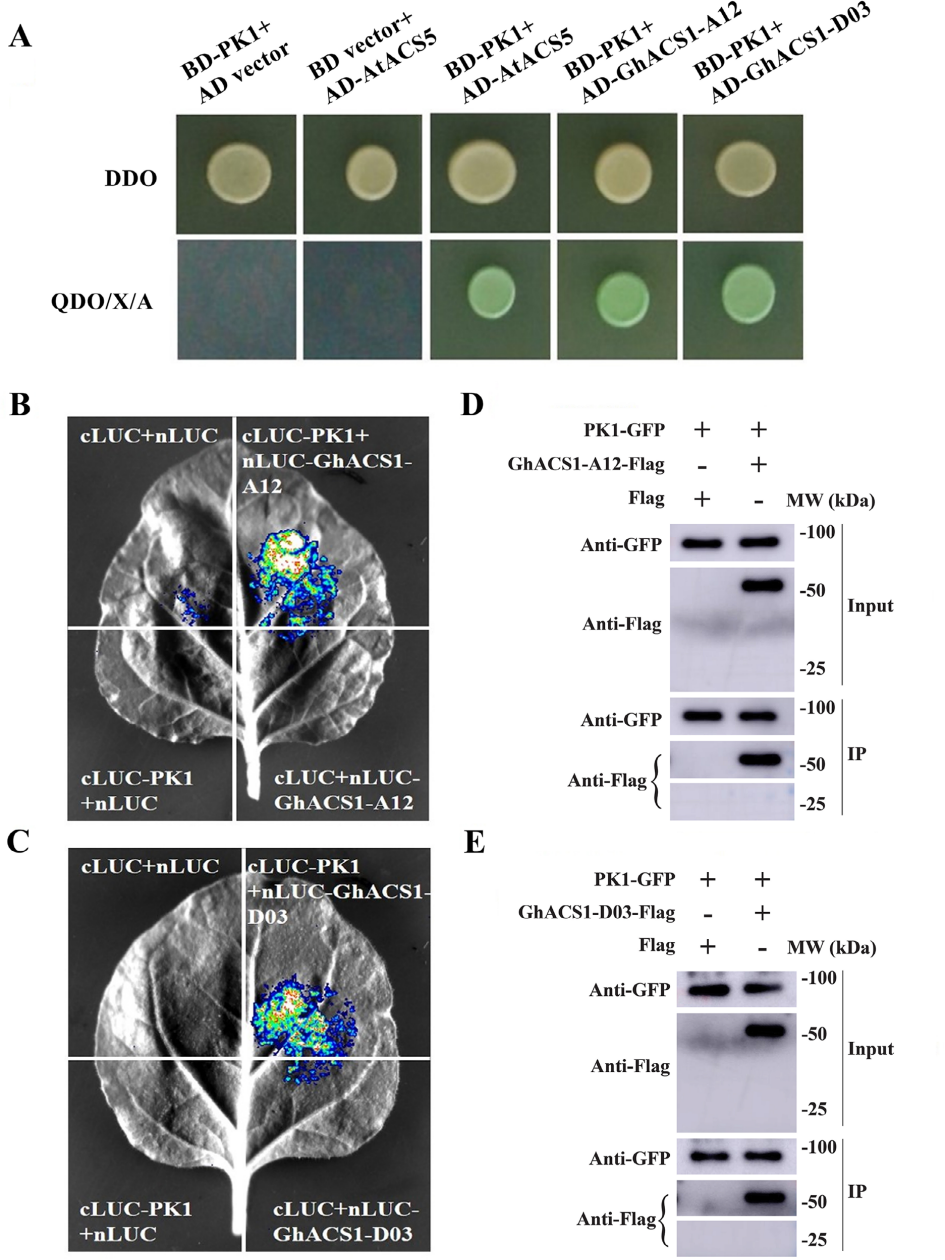
Interactions of PK1 with GhACS1‐A12 and GhACS1‐D03. A) Yeast two‐hybrid assay to investigate the interactions between PK1 and GhACS1‐A12/GhACS1‐D03. Yeast transformants were selected on DDO medium (SD/‐Leu/‐Trp medium) and the higher stringency QDO/X/A medium (SD/‐Ade/‐His/‐Leu/‐Trp with X‐α‐Gal and Aureobasidin A). The negative controls were BD‐PK1+AD vector and BD vector+AD‐AtACS5, while BD‐PK1+AD‐AtACS5 was used as a positive control. B,C) Luciferase complementation imaging assay was performed to detect the interactions between PK1 and GhACS1‐A12 (B) or GhACS1‐D03 (C) in tobacco (*Nicotiana benthamiana*) leaf cells. PK1 was fused to the C‐terminus of the LUC (cLUC) to form cLUC‐PK1, while GhACS1‐A12 and GhACS1‐D03 were fused to the N‐terminus of the LUC (nLUC) to form nLUC‐GhACS1‐A12 and nLUC‐ GhACS1‐D03, respectively. Co‐expression of the truncated LUC and chimeric genes in pairs, as indicated, was carried out in *N. benthamiana* leaves. Negative controls included co‐expression of nLUC and cLUC vectors, nLUC and cLUC‐PK1, cLUC and nLUC‐GhACS1‐A12, cLUC and nLUC‐GhACS1‐D03. D,E) Co‐immunoprecipitation assay was conducted to confirm the interactions between PK1 and GhACS1‐A12 (D) or GhACS1‐D03 (E). To achieve this, co‐immunoprecipitation of PK1‐GFP and GhACS1‐A12/GhACS1‐D03‐Flag transiently co‐expressed in *N. benthamiana* leaves was performed. Protein extracts before (Input) and after immunoprecipitation (IP), and anti‐GFP antibody‐conjugated beads were detected by immunoblotting analysis using an anti‐Flag antibody.

To determine whether Arabidopsis CK1.8^D128N^ (designated PK1) interacts with these cotton ACS proteins, yeast two‐hybrid (Y2H) assays were performed. The results indicated that PK1 specifically interacts with GhACS1‐A12 (Ghicr24_A12G253200) and GhACS1‐D03 (Ghicr24_D03G085500) (Figure **2**A), but not with their direct homoeologs GhACS1‐01926 (Ghicr24_Contig01926G000800) and GhACS1‐A02 (Ghicr24_A02G125300) (Figure S6, Supporting Information). These interactions were further confirmed by luciferase complementation imaging (LCI) and co‐immunoprecipitation (Co‐IP) assays (Figure 2B–E). In support of this selectivity, pairwise sequence comparisons highlight motif‐level differences within the C‐terminal TOE‐like region—LKLSISRSLSRRMVD (GhACS1‐A02) versus LKLSLSRSLSRRMDD (GhACS1‐D03) and LKLSLSRRRDD (GhACS1‐A12) versus LKLSLSRRRGD (GhACS1‐01926)—providing a plausible basis for the observed binding specificity (Figure S7, Supporting Information). Collectively, these results suggest that PK1 can interact with specific cotton ACS proteins, providing a potential molecular target for modulating ethylene biosynthesis in cotton fibers.

### 2.4 Ethylene Dose‐Dependently Regulates Fiber Elongation and SCW Deposition

**Figure 3:**
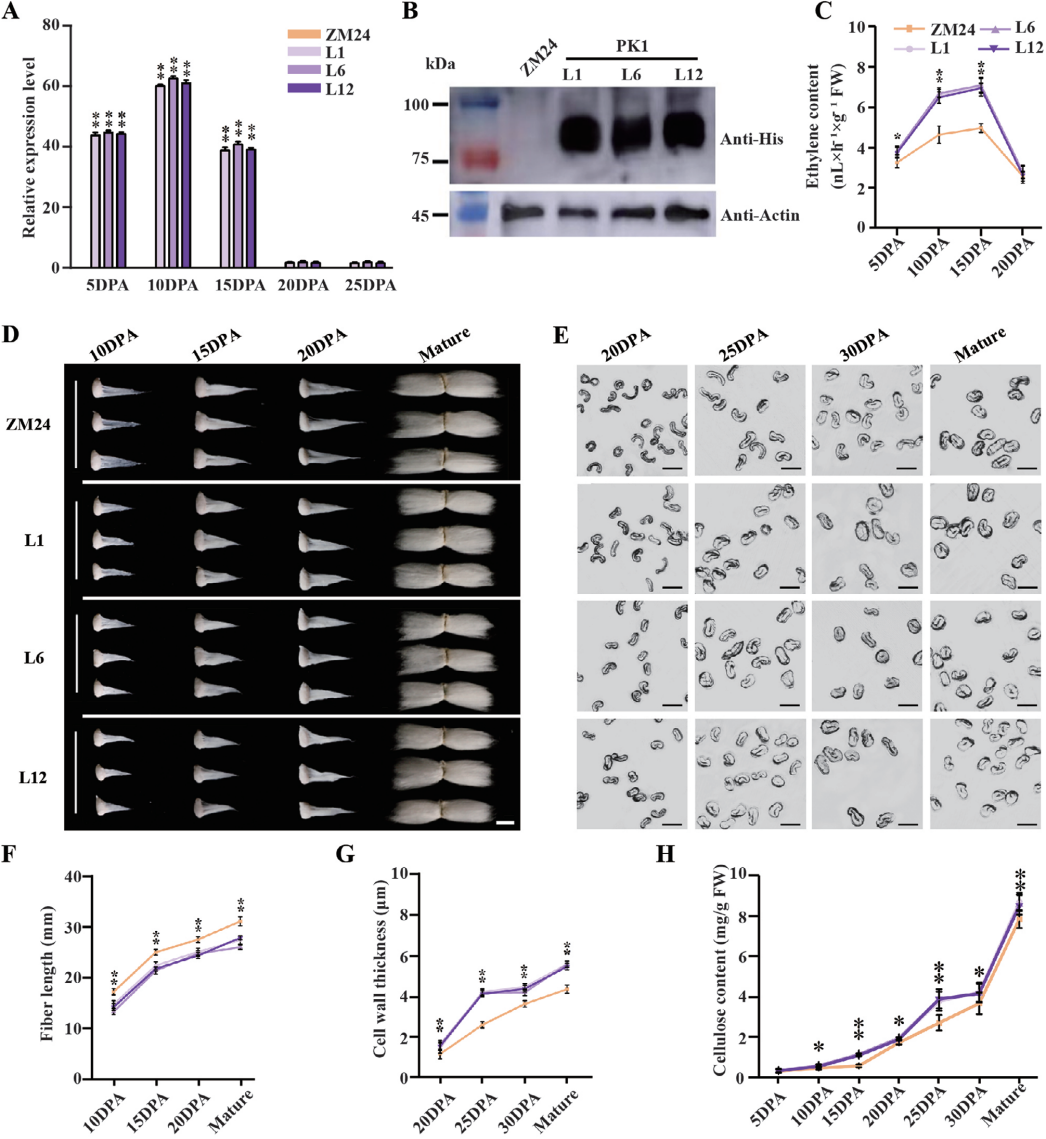
Identification and fiber characteristic analysis of *PK1* transgenic lines. A) Quantitative real‐time RT‐PCR (qRT‐PCR) was conducted to analyze the expression levels of *PK1* in fibers at different developmental stages of ZM24 and *PK1* transgenic lines. Values are presented as means ± SD of triplicate assays. B) Immunoblot analysis was performed to detect *PK1* protein expression in 10 DPA fibers of ZM24 and *PK1* transgenic lines using a His‐tagged antibody, with β‐actin as an internal reference. C) The ethylene content in fibers of ZM24 and *PK1* transgenic lines at different developmental stages was measured, with at least 30 fibers measured per replicate. D) Representative images of fibers from ZM24 and *PK1* transgenic lines at different developmental stages are presented. Bar = 1 cm. E) Cross‐sectional images of fibers from ZM24 and *PK1* transgenic lines at different developmental stages. Bar = 10 µm. F) Measurement and statistical analysis of fiber length of ZM24 and *PK1* transgenic lines at distinct developmental stages (n > 30). G) Cell wall thickness of fibers at various developmental stages from ZM24 and *PK1* transgenic lines (n > 30). H) Cellulose content of fibers at 5, 10, 15, 20, 25, 30 DPA and mature stages from three biological replicates of ZM24 and PK1 transgenic lines was measured. Mean values and standard deviations of three biological replicates are presented. Student's t‐tests confirmed significant differences (^*^, *P* < 0.05; ^**^, *P* < 0.01) between the ZM24 and the *PK1* transgenic lines. ZM24 is the wild‐type plant, while L1, L6, and L12 denote three independent lines of *PK1* transgenic cotton (*Gossypium hirsutum*).

To investigate the effects of altered endogenous ethylene levels on cotton fiber development, we first sought to increase its production. We generated transgenic cotton (*G. hirsutum* cv. ZM24) overexpressing *PK1* under the control of the fiber‐specific *GhRDL1* promoter. Successful overexpression in multiple independent lines was confirmed at both the transcript and protein levels during the fiber elongation phase (5‐15 DPA) (Figure **3**A,B). Consistent with the *PK1* expression pattern, these *PK1* OE lines exhibited significantly elevated ethylene content in developing fibers. The increases relative to ZM24 were substantial: 15.2–17.9% at 5 DPA, 40.0–44.4% at 10 DPA, and 40.0–43.0% at 15 DPA across three independent lines (Figure 3C). Importantly, these genetic modifications did not affect overall plant architecture, confirming the fiber‐specific nature of the promoter activity (Figure S8, Supporting Information).

This engineered elevation in ethylene had a significant impact on fiber quality. Over three years, mature fibers from *PK1* OE lines were consistently shorter but significantly stronger than those from ZM24, with minimal changes to micronaire (Table S6, Supporting Information). A detailed developmental analysis revealed that transgenic lines had reduced fiber length at 10, 15, 20 DPA, and the mature stage (Figure 3D,F), but showed markedly increased SCW thickness at 20, 25, 30 DPA, and maturity (Figure 3E,G). Consistently, cellulose content in transgenic fibers was significantly higher from 10 DPA onward, with a notable early increase at 10 and 15 DPA, suggesting accelerated SCW deposition (Figure 3H).

**Figure 4:**
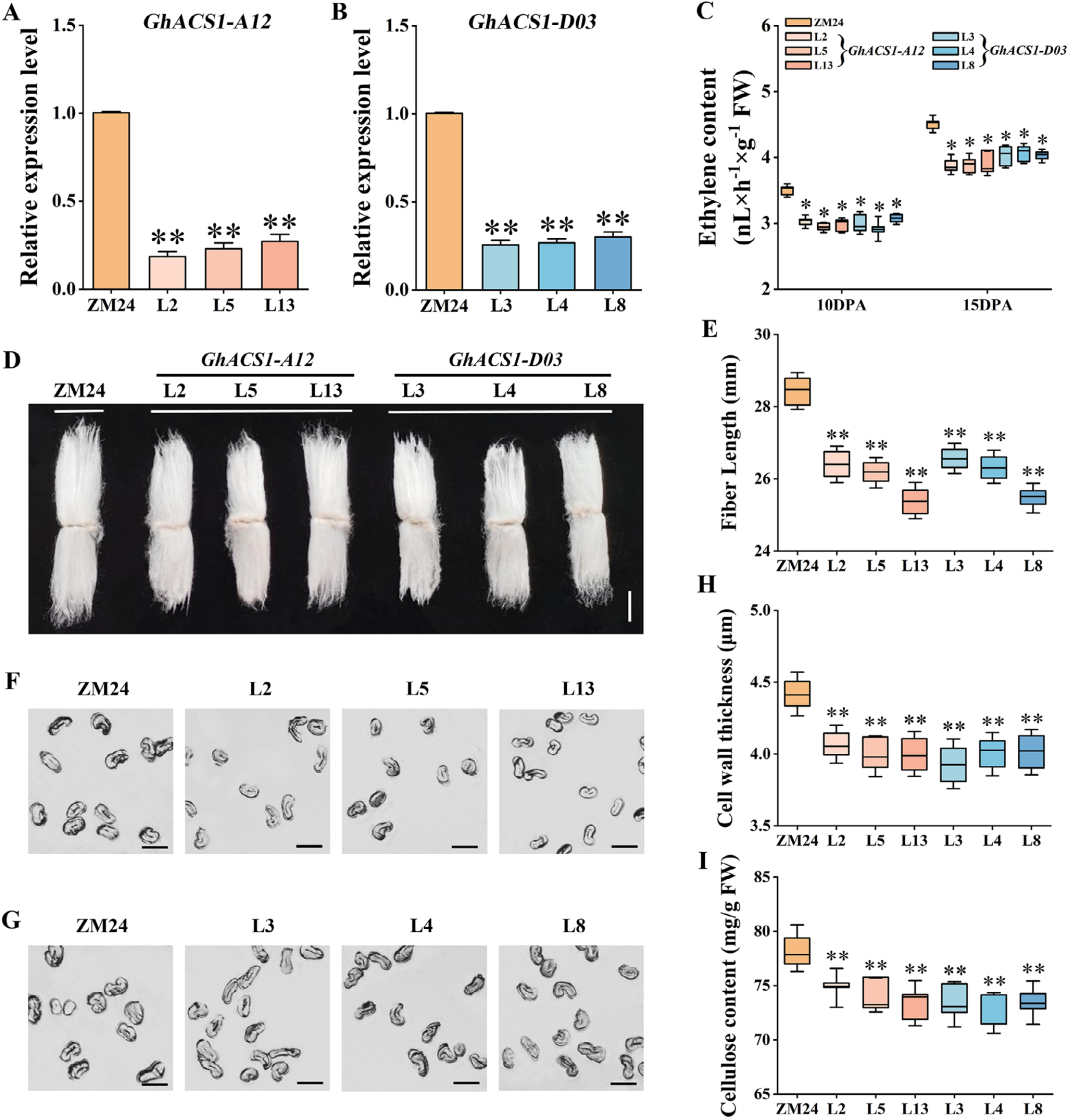
Identification and fiber characteristic analysis of *GhACS1‐A12* and *GhACS1‐D03* RNA interference (RNAi) lines. A,B) qRT‐PCR was performed to assess the expression levels of *GhACS1‐A12* (A) and *GhACS1‐D03* (B) in 10 DPA fibers of ZM24, as well as *GhACS1‐A12* and *GhACS1‐D03* RNAi lines. Data are presented as means ± SD of triplicate assays. C) The ethylene content in 10 and 15 DPA fibers of ZM24, as well as *GhACS1‐A12* and *GhACS1‐D03* RNAi lines was measured, with at least 30 fibers measured per replicate. D) Representative mature cotton fibers of the ZM24 as well as *GhACS1‐A12* and *GhACS1‐D03* RNAi lines. Bar = 1 cm. E) Statistical analysis of fiber length of the ZM24 as well as *GhACS1‐A12* and *GhACS1‐D03* RNAi lines. Error bars indicate SD (n > 30). F,G) Cross‐sectional images of mature fibers from ZM24 as well as *GhACS1‐A12* and *GhACS1‐D03* RNAi lines. Bar = 10 µm. H) Cell wall thickness of mature fibers from ZM24, *GhACS1‐A12*, and *GhACS1‐D03* RNAi lines (n > 30). I) Cellulose content of mature fibers from three biological replicates of ZM24, *GhACS1‐A12*, and *GhACS1‐D03* RNAi lines. Mean values and standard deviation of three biological replicates are presented. Independent *t*‐tests demonstrated that there were significant differences (^*^, ***P*** < 0.05; ^**^, *P* < 0.01) between the ZM24 and the *GhACS1‐A12* RNAi lines, as well as between the ZM24 and the *GhACS1‐D03* RNAi lines. ZM24 is the wild‐type plant, while L2, L5, and L13 are *GhACS1‐A12* RNAi lines and L3, L4, and L8 are *GhACS1‐D03* RNAi lines.

Conversely, to assess the impact of reduced ethylene, we suppressed its endogenous biosynthesis by generating RNAi lines targeting two key *GhACS1* homologs (*GhACS1‐A12* and *GhACS1‐D03*). qRT‐PCR confirmed significant down‐regulation of both target genes in the respective RNAi lines (Figure **4**A,B). To assess RNAi specificity, we quantified transcripts of the other six *ACS* genes that are highly expressed in fibers; none showed significant expression changes in either *GhACS1‐A12* RNAi or *GhACS1‐D03* RNAi lines relative to ZM24 (Figure S9, Supporting Information). This led to a corresponding and significant decrease in fiber ethylene content. At 10 DPA, ethylene levels were reduced by 13.5–16.1% and 12.4–16.7% in *GhACS1‐A12* and *GhACS1‐D03* RNAi lines, respectively. This reduction was maintained at 15 DPA (13.2–13.7% and 10.0–11.0%, respectively) (Figure 4C). While the RNAi plants exhibited a slight reduction in overall plant height (Figure S10, Supporting Information), the most striking effects were observed in fiber development. This ethylene reduction resulted in significantly shorter mature fibers (Figure 4D,E). However, in stark contrast to the *PK1* OE lines, these fibers had thinner SCWs and markedly reduced cellulose content compared to ZM24 (Figure 4F–I). Pharmacological tests on ovules from ZM24, *PK1* OE, and *GhACS1‐D03* RNAi lines confirmed ethylene's dose‐dependent effect on fiber elongation. Specifically, fibers from *PK1* OE were significantly longer than ZM24 under treatment with the ethylene synthesis inhibitor AVG, while the opposite was observed with the precursor ACC; conversely, in the *GhACS1‐D03* RNAi lines, AVG treatment exacerbated the short‐fiber phenotype, whereas ACC application partially restored fiber length (Figure S11, Supporting Information). Taken together, these results demonstrate that ethylene orchestrates fiber development in a complex, dose‐dependent manner. An optimal, intermediate level of ethylene is critical for achieving maximal fiber elongation. In contrast, ethylene appears to act as a monotonic promoter of SCW biosynthesis within the physiological range tested. Consequently, elevating ethylene above the elongation optimum mediates a developmental trade‐off—enhancing fiber strength while compromising length—whereas ethylene deficiency reduces both traits.

### 2.5 Altered Stability of GhACS1 Isoforms Influences Ethylene Biosynthesis in Upland Cotton Fibers

To investigate the mechanisms underlying increased ethylene levels in *PK1* OE lines, we examined the transcript and protein abundance of GhACS1‐A12 and GhACS1‐D03, two key ethylene biosynthetic enzymes that interact with PK1 (Figure 2). Transcript analysis showed no significant difference in *GhACS1‐A12* and *GhACS1‐D03* mRNA levels between ZM24 and transgenic lines (Figure S12A, Supporting Information), suggesting post‐transcriptional regulation. Proteasome inhibition assays revealed that both GhACS1‐A12 and GhACS1‐D03 proteins accumulated significantly following MG132 treatment in 10 DPA fibers, confirming that their stability is regulated by the ubiquitin‐proteasome pathway. Importantly, protein levels of both enzymes were consistently higher in transgenic lines than in ZM24, regardless of MG132 treatment (Figure S12B, Supporting Information). Furthermore, in vivo ubiquitination assays on 10 DPA fibers showed that polyubiquitination of both GhACS1‐A12 and GhACS1‐D03 proteins was markedly reduced in *PK1* OE lines compared with ZM24 (Figure S12C, Supporting Information). Together, these data indicate that PK1 enhances the stability of GhACS1 isoforms by diminishing their ubiquitination and proteasomal turnover, thereby promoting ethylene biosynthesis in cotton fibers.

### 2.6 Transcriptome Analysis Reveals Ethylene‐Mediated Regulation of Fiber Development

To elucidate the molecular mechanisms underlying ethylene‐mediated fiber development, transcriptome sequencing was performed on fibers collected from ZM24 (wild‐type, WT), *PK1* overexpression (ethylene overproduction, EO), and *GhACS1‐D03* RNAi (ethylene deficient, ED) lines at 10 and 15 DPA. Differential expression analysis identified 518 (EO vs WT) and 704 (ED vs WT) DEGs at 10 DPA, and 513 (EO vs WT) and 682 (ED vs WT) DEGs at 15 DPA (Figure S13A,B, Supporting Information). The gene ontology (GO) enrichment analysis showed significant enrichment of genes in ethylene signaling, SCW biogenesis, and cell wall polysaccharide metabolism (Figure S13C, Supporting Information).

**Figure 5:**
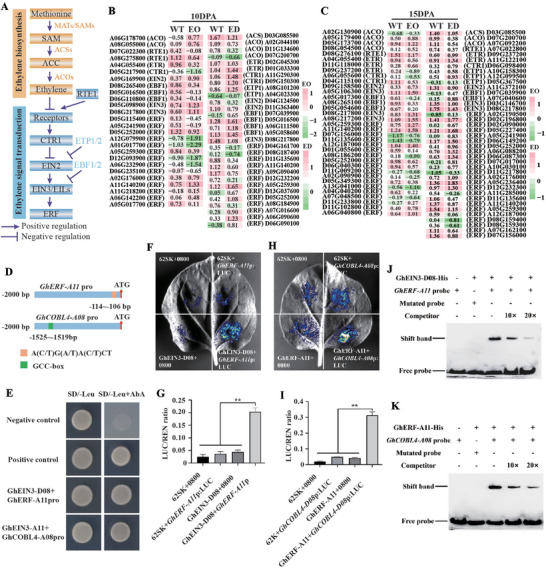
Ethylene signaling cascade and the GhEIN3‐GhERF‐GhCOBL4 transcriptional module. A) Diagram of ethylene biosynthesis and signaling. Solid arrows, positive regulation; bar‐ended arrows, negative regulation. B,C) Heat‐maps of ethylene‐associated genes in ethylene overproduction (EO) and ethylene deficient (ED) plants at 10 DPA (B) and 15 DPA (C). The gene IDs shown here are prefixed with Ghicr24. D) Promoter fragments used for protein‐DNA interaction assays. A putative EIN3‐binding site in *GhERF‐A11* promoter and a GCC‐box in *GhCOBL4‐A08* promoter are indicated. E) Yeast one‐hybrid assays. GhEIN3‐D08 binds *GhERF‐A11* promoter, and GhERF‐A11 binds *GhCOBL4‐A08* promoter. Yeast was grown on SD/‐Leu (control) or SD/‐Leu + AbA (selection). F,G) Dual‐luciferase assay in *Nicotiana benthamiana* leaves showing activation of *GhERF‐A11* promoter by GhEIN3‐D08. Representative luminescence images. 62SK is the effector plasmid pGreen II 62‐SK, and 0800 is the reporter plasmid pGreen II 0800‐LUC (F). LUC/REN ratios (mean ± SD, n = 3). ^**^ *P* < 0.01 (Student's *t*‐test). The expression of REN is used as an internal control (G). H,I) Dual‐luciferase assay showing activation of *GhCOBL4‐A08* promoter by GhERF‐A11. Data presentation as in (F,G). J) Electrophoretic mobility shift assay confirming direct binding of GhEIN3‐D08 to the *GhERF‐A11* promoter probe. FAM (carboxyfluorescein)‐labeled probes were incubated with His‐GhEIN3‐D8 in vitro. Unlabeled probes were used for competition, and FAM‐labeled mutated probes were used as a negative control. K) EMSA showing direct binding of GhERF‐A11 to the *GhCOBL4‐A08* promoter GCC‐box. Competition assays and negative control as in (J).

Focusing on ethylene biosynthesis and signaling, 61 and 69 DEGs were identified in the EO versus WT and ED versus WT comparisons, respectively (Figure **5**A–C; Tables S7–S14, Supporting Information). In EO lines, key ethylene biosynthetic enzymes (e.g., *ACO*) and positive regulators (e.g., *EIN2*, *EIN3*) were up‐regulated, while negative regulators (e.g., *ETR*, *CTR1*, *EIN3‐binding F‐box protein 1* [*EBF1*]) were down‐regulated. The opposite pattern was observed in ED lines. Numerous differentially expressed *ethylene response factors* (*ERFs*) were also detected in both comparisons.

Analysis of downstream targets revealed that cytoskeletal genes (e.g., *tubulin*, *actin‐related protein* [*ARP*], *microtubule‐associated protein* [*MAP*]) associated with fiber elongation were up‐regulated in EO versus WT but down‐regulated in ED versus WT. Most *EXPANSIN* and *fasciclin‐like arabinogalactan protein* (*FLA*) genes were down‐regulated in both comparisons. For cell wall biosynthesis, genes encoding cellulose synthase (*CESA*) and lignin biosynthesis enzymes (*4‐coumarate‐CoA ligase* [*4CL*], *caffeoyl‐CoA O‐methyltransferase* [*COMT*], *cinnamyl alcohol dehydrogenase* [*CAD*]) were up‐regulated in EO versus WT and down‐regulated in ED versus WT. In contrast, genes involved in xylan metabolism (*xylosidase* [*XYL*], *irregular xylem* [*IRX*], *glucuronic acid substitution of xylan* [*GUX*]) were up‐regulated in both lines. Genes related to xyloglucan modification (*xyloglucan endotransglucosylase/hydrolase protein* [*XTH*], *xyloglucan glycosyltransferase*) were down‐regulated in EO versus WT but up‐regulated in ED versus WT. Similarly, genes involved in pectin metabolism (*pectate lyase*, *pectinesterase*, *pectinesterase inhibitor*) showed a balanced regulation in EO versus WT but were predominantly up‐regulated in ED versus WT (Figure S14, Supporting Information). The transcriptome results were validated by qRT‐PCR analysis (Figure S15, Supporting Information). Collectively, these findings indicate that ethylene exerts a complex and multifaceted influence on cotton fiber development by modulating both early elongation and subsequent cell wall biosynthesis.

### 2.7 Ethylene Signaling Orchestrates a Transcriptional Cascade Governing Upland Cotton Fiber Development

To clarify the role of ethylene signaling in fiber development, we analyzed gene expression differences between WT, EO, and ED lines, focusing on nine DEGs consistently regulated across developmental stages in both EO versus WT and ED versus WT comparisons (Figure S13B, Supporting Information). These included the transcription factor *GhEIN3‐D08*, three *ERF* family members (*GhERF‐A05*, *GhERF‐D05*, *GhERF‐A11*), and genes involved in cell wall biosynthesis and modification (*GhCOBL4‐A08*, *GhCESA4‐D07*), protein ubiquitination (*GhUBI‐A10*), proteolysis (*GhCEP‐A03*), and lipid metabolism (*GhGPAT‐A09*). Expression profiling revealed that *GhEIN3‐D08*, *GhERF‐A11*, *GhCOBL4‐A08*, and *GhCESA4‐D07* were up‐regulated in EO versus WT and down‐regulated in ED versus WT, while *GhERF‐D05* and *GhUBI‐A10* exhibited the opposite pattern. The remaining genes showed unique or similar trends (Figure S16, Supporting Information).

To elucidate the regulatory mechanisms, we conducted in silico promoter analysis, identifying EIN3‐binding motifs [A(C/T)G(A/T)A(C/T)CT] in the promoters of *GhERF‐A05*, *GhERF‐A11*, and *GhUBI‐A10*, and ERF‐binding motifs (GCC‐box) in *GhCOBL4‐A08*. Yeast one‐hybrid (Y1H) assays confirmed the direct binding of GhEIN3‐D08 to the *GhERF‐A11* promoter, and of GhERF‐A11 to the *GhCOBL4‐A08* promoter (Figure 5D,E; Figure S17, Supporting Information). These interactions were further validated by dual‐luciferase reporter and electrophoretic mobility shift assays (Figure 5F–K). Taken together, these results support a hierarchical transcriptional cascade in which ethylene signaling, via GhEIN3‐D08, directly activates *GhERF‐A11*, which subsequently regulates *GhCOBL4‐A08*. This regulatory module highlights the central role of ethylene in orchestrating gene expression networks essential for cotton fiber development.

### 2.8 Elevated Ethylene Elicits an Opposite Phenotypic and Transcriptional Response in *G. arboreum*

**Figure 6:**
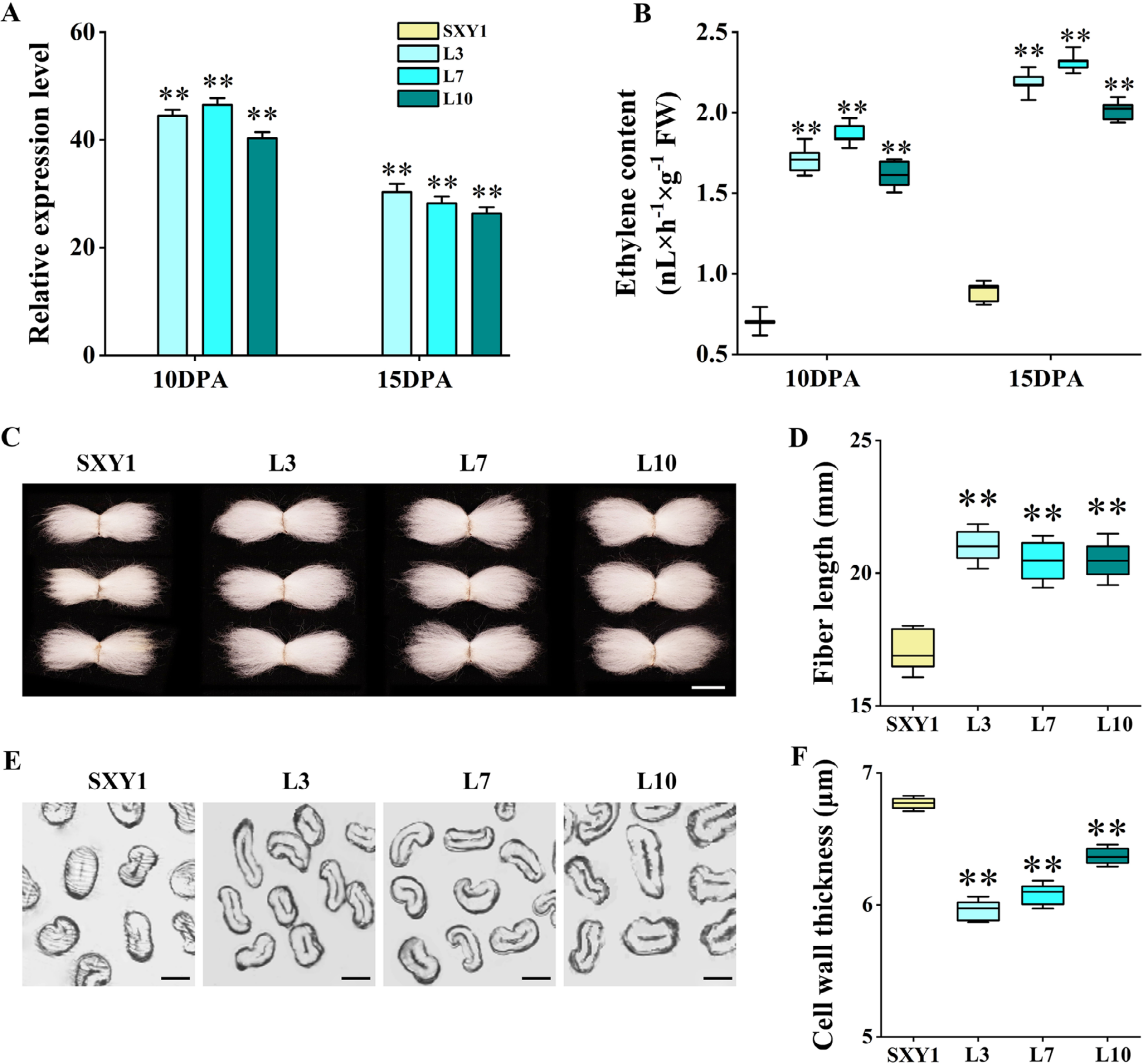
Fiber development and ethylene production in *Gossypium arboreum* (SXY1). A) Quantitative real‐time RT‐PCR (qRT‐PCR) was conducted to analyze the expression levels of *PK1* in fibers at different developmental stages of SXY1 and *PK1* overexpression lines (mean ± SD, n = 3). B) The ethylene content in fibers of SXY1 and *PK1* overexpression lines at different developmental stages was measured, with at least 30 fibers measured per replicate. C) Representative mature cotton fibers of the SXY1 and *PK1* overexpression lines. Bar = 1 cm. D) Statistical analysis of fiber length of the SXY1 and *PK1* overexpression lines. Error bars indicate SD (n > 30). E) Cross‐sectional images of mature fibers from SXY1 and *PK1* overexpression lines. Bar = 10 µm. F) Cell wall thickness of mature fibers from SXY1 and *PK1* overexpression lines (n > 30). Student's *t*‐tests showed that the differences between the SXY1 and *PK1* overexpression lines were statistically significant (^**^, *P* < 0.01). SXY1 is the wild‐type plant, while L3, L7, and L10 denote three independent lines of *PK1* overexpression transgenic cotton (*Gossypium arboreum*).

To investigate whether the dose‐dependent effects of ethylene are conserved across species, we generated *G. arboreum* cv. SXY1 transgenic lines overexpressing *PK1* under the *GhRDL1* promoter (*G. raimondii* currently lacks a reliable *Agrobacterium*‐mediated transformation system). High *PK1* expression was confirmed in transgenic fibers during the elongation stage (**Figure** **6**A), leading to a significant increase in ethylene production. At 10 and 15 DPA, ethylene levels in three independent transgenic lines were markedly higher (127.8–165.4% and 128.5–163.3%, respectively) than in the SXY1 (Figure 6B). Notably, unlike in *G. hirsutum*, this engineered increase in ethylene resulted in substantially longer mature fibers with markedly thinner SCW (Figure 6C–F). *PK1* overexpression in SXY1 did not alter plant height and other morphology; however, leaves displayed a modest reduction in blade area (Figure S18, Supporting Information). These findings demonstrate that in the *G. arboreum* background, elevated ethylene promotes fiber elongation at the expense of SCW deposition.

To determine if the EIN3‐ERF‐COBL4 cascade from *G. hirsutum* was inherited from its diploid ancestor, we investigated this regulatory module in *G. arboreum*. We identified the orthologs of *GhEIN3‐D08*, *GhERF‐A11*, and *GhCOBL4‐A08* in *G. arboreum*. Subsequent yeast one‐hybrid (Y1H) assays confirmed that the regulatory cascade is conserved: GaEIN3‐08 binds directly to the *GaERF‐11* promoter, and GaERF‐11 binds to the *GaCOBL4‐08* promoter (Figure S19A, Supporting Information). However, the transcriptional response of this module to ethylene was inverted. In contrast to the up‐regulation observed in *G. hirsutum PK1* OE lines, the expression of *GaEIN3‐08*, *GaERF‐11*, and *GaCOBL4‐08* was significantly down‐regulated in *G. arboreum PK1* OE fibers at both 10 and 15 DPA (Figure S19B–D, Supporting Information). This finding suggests that while the EIN3‐ERF‐COBL4 regulatory module is an ancient and conserved feature, its downstream transcriptional response to ethylene signaling has diverged between the two species, providing a molecular explanation for their distinct fiber developmental morphology.

## 3 Discussion

**Figure 7:**
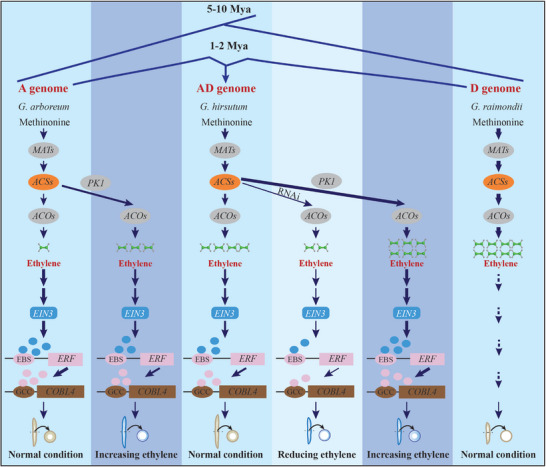
A working model for the dose‐dependent and evolutionarily divergent regulation of cotton fiber development by the ethylene‐EIN3‐ERF‐COBL4 module. The model illustrates the ethylene biosynthesis and signaling pathway and its functional consequences on fiber development in allotetraploid *G. hirsutum* (AD genome) and its diploid ancestor *G. arboreum* (A genome), placed within the context of their evolutionary divergence from the D‐genome ancestor, *G. raimondii*. (Left panels, *G. arboreum*) Under normal conditions, *G. arboreum* exhibits low endogenous ethylene levels, which leads to a de‐repression of the downstream transcriptional cascade, resulting in thicker SCWs. Increasing ethylene levels via PK1‐mediated stabilization of ACS isoforms triggers a repressive signal, leading to the downregulation of the EIN3‐ERF‐COBL4 cascade. This functional inversion promotes fiber elongation at the expense of SCW deposition, yielding longer, thinner fibers. (Center panels, *G. hirsutum*) Under normal conditions, *G. hirsutum* maintains an optimal, intermediate ethylene level that supports maximal fiber elongation. Reducing ethylene via RNAi‐mediated suppression of *GhACS1* impairs the activation of the EIN3‐ERF‐COBL4 cascade, resulting in fibers that are both shorter and have thinner SCWs. Conversely, increasing ethylene via *PK1* overexpression leads to hyper‐activation of the cascade, promoting SCW deposition but prematurely halting elongation, which results in a “short‐but‐strong” fiber phenotype. This demonstrates a dose‐dependent effect and a regime‐specific balance between fiber length and strength: elevating ethylene above the elongation optimum yields shorter but stronger fibers, whereas lowering ethylene reduces both traits. (Right panel, *G. raimondii*) The D‐genome ancestor exhibits naturally high levels of ethylene, which correlate with the shortest and thinnest fibers, suggesting its developmental program is distinct from the other species. The relative abundance of molecules (ethylene) and protein complexes (blue EIN3 circles, pink ERF circles) visually represents their concentration or activity levels. Within each species panel, the thickness of arrow‐headed lines indicates the relative strength of promotion for ethylene synthesis and signal transduction; thicker lines denote stronger promotion, while thinner lines denote weaker promotion. The final fiber phenotype illustrates changes in length and SCW thickness. This model highlights how the functional rewiring of a physically conserved transcriptional cascade following polyploidization provides a molecular basis for species‐specific fiber traits and the dose‐dependent balance between fiber length and strength.

In this study, we decipher a complete regulatory axis, from the post‐translational control of ethylene biosynthesis to a downstream transcriptional cascade, that quantitatively governs the dose‐dependent balance between cotton fiber elongation and strength. We provide definitive genetic evidence that the dose‐dependent effects of ethylene, a long‐hypothesized but mechanistically obscure phenomenon, are orchestrated by a hierarchical EIN3‐ERF‐COBL4 module. Most remarkably, we reveal that this entire regulatory system has undergone profound functional divergence following polyploidization, providing a molecular explanation for the distinct fiber characteristics across *Gossypium* species and offering novel strategies to uncouple two of the most critical traits in cotton improvement (**Figure** **7**).

Our work first establishes a direct link between the natural variation in ethylene content and the divergent fiber traits of *G. hirsutum* and its diploid progenitors, *G. arboreum* and *G. raimondii* (Figure 1). This observation challenges the simplistic notion of ethylene as a universal promoter of elongation and instead supports a more nuanced, dose‐dependent model. Maximal fiber elongation in tetraploid cotton requires an optimal, intermediate ethylene concentration, as both elevated and reduced levels are inhibitory. This biphasic response is a classic feature of hormonal regulation, ensuring developmental processes are maintained within a homeostatic range.^[^ ^24^, ^25^ ^]^ Our findings suggest that the superior fiber length of *G. hirsutum* is not merely an additive trait but an emergent property of a rewired ethylene signaling network, established after the merger of the A and D sub‐genomes, which achieved a new, optimal set point for ethylene concentration.

A key mechanistic advance is our identification of the PK1‐ACS1 module as an upstream control point. By demonstrating that a kinase‐deficient CK1 variant (PK1) stabilizes specific, highly expressed type‐I ACS isoforms (*GhACS1‐A12* and *GhACS1‐D03*) at the protein level (Figure S11, Supporting Information), we uncover a critical post‐translational mechanism for fine‐tuning ethylene output during fiber development. Consistent with this selective mode of action, PK1 fails to bind the direct homoeologs GhACS1‐01926 and GhACS1‐A02 (Figure S6, Supporting Information). Both homoeologous pairs differ specifically within the C‐terminal TOE‐like region, encompassing an (F/L)RLS(F/L)‐like motif and an adjacent acidic R/D/E‐rich segment (Figure S7, Supporting Information). Because these short motifs encode regulator‐binding and kinase‐responsive functions in ACS proteins,^[^ ^26^, ^27^, ^28^ ^]^ we propose that isoform‐specific features of this C‐terminal zone dictate PK1 binding and thereby explain the selective stabilization of GhACS1‐A12 and GhACS1‐D03. This finding parallels regulatory systems in other species^[^ ^17^ ^]^ but reveals a novel target specificity, highlighting how conserved kinases can be repurposed to regulate distinct substrates and, consequently, unique developmental programs.

To further substantiate the mechanism by which PK1 stabilizes ACS, we show that PK1 reduces in vivo polyubiquitination of GhACS1‐A12 and GhACS1‐D03 in fibers (Figure S12C, Supporting Information). Together with unchanged *GhACS1‐A12* and *GhACS1‐D03* transcripts (Figure S12A, Supporting Information) and increased ACS1 protein accumulation upon proteasome inhibition, with *PK1* OE lines retaining higher ACS1 levels regardless of MG132 treatment (Figure S12B, Supporting Information), these data support a model in which PK1 limits ubiquitin‐proteasome‐dependent turnover of ACS1 isoforms. Given that PK1 is a kinase‐deficient CK1.8 variant,^[^ ^17^ ^]^ we propose that PK1 acts as a substrate‐protective scaffold at the ACS1 C terminus, masking degron features within the TOE‐like region and thereby reducing access by ACS‐targeting E3 ubiquitin ligase complexes.^[^ ^17^, ^18^ ^]^ The observed binding selectivity—PK1 associates with GhACS1‐A12 and GhACS1‐D03 but not their close homoeologs that differ in the C‐terminal TOE‐like motifs—supports this model. Identifying the cognate cotton E3 ligase(s) and mapping ACS1 ubiquitination sites, together with in vitro ubiquitination reconstitution in the presence and absence of PK1, are clear next steps to refine this mechanism.

Downstream of the signal, we uncover a linear transcriptional cascade that translates ethylene dosage into a developmental outcome. The direct activation of *GhERF‐A11* by GhEIN3‐D08, which in turn binds to and regulates the promoter of *GhCOBL4‐A08*, provides a complete molecular framework connecting the master ethylene signaling pathway to a key effector of cellulose microfibril deposition (Figure 5). *COBRA‐LIKE* genes are critical for cell wall integrity and anisotropic growth.^[^ ^29^, ^30^ ^]^ By linking EIN3 directly to COBL4 via an intermediary ERF, our model explains how ethylene can precisely coordinate the timing and extent of SCW deposition. The transcriptome data further support this, showing that elevated ethylene (in *PK1* OE lines) activates a suite of SCW‐related genes while repressing genes associated with cell wall expansion (Figure S13, Supporting Information). This premature shift from elongation to thickening explains the “short‐but‐strong” fiber phenotype and mechanistically underpins the length‐strength trade‐off.

Perhaps our most significant discovery is the functional divergence of this entire regulatory module. In stark contrast to *G. hirsutum*, elevating ethylene in the diploid ancestor *G. arboreum* produced the opposite phenotype: longer, thinner fibers (Figure 6). This result is explained by our finding that the transcriptional response of the EIN3‐ERF‐COBL4 cascade, while physically conserved, is functionally inverted (Figure S18, Supporting Information). Elevated ethylene activates the cascade in *G. hirsutum* but represses it in *G. arboreum* (Figure 7). This rewiring of a conserved signaling module is a profound evolutionary insight. It suggests that the naturally low ethylene levels in *G. arboreum* may lead to a de‐repression of this cascade, driving the formation of its characteristic thick SCWs. We speculate that this inverted logic is adaptive in several, non‐exclusive ways: by coupling high ethylene—an integrator of heat, drought, and biotic stress—to repression of SCW deposition, *G. arboreum* may reduce the carbon cost of cellulose synthesis while permitting continued elongation, thereby prioritizing embryo provisioning under stress;^[^ ^5^, ^31^, ^32^ ^]^ longer, finer fibers under elevated ethylene may enhance seed dispersal via greater aerodynamic loft and potential zoochory, advantageous in the seasonally variable Old World habitats where A‐genome species evolved;^[^ ^33^, ^34^ ^]^ and repressing SCW when ethylene is high could delay wall stiffening late in development, extending the elongation window and buffering against environmental fluctuations.^[^ ^35^, ^36^ ^]^ By contrast, in *G. hirsutum* ethylene activates the EIN3–ERF–COBL4 cascade, a configuration likely established during polyploidization and retained under domestication to favor SCW deposition and fiber strength essential for textile performance.^[^ ^33^, ^37^ ^]^

What mechanisms might underlie this inversion? Although the physical cascade is conserved (Figure S19A, Supporting Information), elevated ethylene represses these nodes in *G. arboreum* (Figure S19B–D, Supporting Information), whereas it activates the orthologous module in *G. hirsutum* (Figure 5). We propose three non‐mutually exclusive explanations. First, *cis*‐regulatory rewiring at *GaEIN3‐08*, *GaERF‐11*, and *GaCOBL4‐08* promoters may convert transcription factor occupancy into repression: changes in the number, spacing, and orientation of these elements, or the presence of additional flanking motifs, could favor recruitment of co‐repressors in *G. arboreum*.^[^ ^38^ ^]^ Second, *trans*‐acting differences could invert output polarity: sequence divergence in *GaERF‐11* (e.g., in activation/repression domains or potential EAR‐like motifs), altered post‐translational modification of EIN3 or ERF proteins, or species‐specific availability of cofactors (e.g., TOPLESS/HDAC complexes or Mediator subunits) could shift these factors from activators to repressors under ethylene.^[^ ^39^, ^40^, ^41^, ^42^ ^]^ Third, epigenetic and network‐level context may differ between species: ethylene could engage distinct chromatin states at *ERF*/*COBL4* loci (e.g., changes in H3K27ac/H3K27me3 or accessibility), or stronger negative feedback through ETR/CTR1/EBF kinetics could bias the steady state toward repression in *G. arboreum*.^[^ ^43^, ^44^ ^]^ Together, these hypotheses provide a mechanistic framework for explaining how a physically conserved module can be functionally inverted across *Gossypium* lineages.

In conclusion, our study provides a comprehensive, multi‐layered understanding of ethylene's role in fiber development. We establish a molecular framework for its dose‐dependent effects, identify the upstream and downstream regulatory components, and reveal its evolutionary plasticity. This work transforms our view of the length‐strength trade‐off from an immutable linkage to a tunable, hormone‐regulated developmental program. The identification of the functionally divergent EIN3‐ERF‐COBL4 module not only deepens our understanding of hormone signaling evolution but also provides a toolkit of precise molecular targets. Engineering the expression or regulation of these specific cascade components, rather than global ethylene levels, offers a sophisticated and promising strategy to finally break the negative correlation between fiber length and strength, paving the way for the next generation of elite cotton varieties.

## 4 Experimental Section

### Cotton Materials

Seeds of the ZM24 cultivar of upland cotton (*G. hirsutum*), Shixiya1 (SXY1) cultivar of *G. arboreum*, and D_5_‐3 cultivar of *G. raimondii* were subjected to surface sterilization using 3% (v/v) H_2_O_2_ for 24 h, followed by washing with sterile water. The sterilized seeds were germinated on Murashige and Skoog (MS) medium under a 16‐h light/8‐h dark cycle at 28 °C for one week. The sterile seedlings were then transplanted into soil and grown to maturity. These mature plants were then used for further experiments.

### Construction of Vector and Genetic Transformation of Cotton Plants

To generate the overexpression vector, the coding sequence of Arabidopsis *CK1.8^D128N^* was amplified by fusion PCR and subcloned into the pCambia1302 vector;^[^ ^17^ ^]^ to this heterologous kinase‐deficient protein was referred to as PK1. For construction of the RNA interference (RNAi) vector, a 260 bp specific sequence of the *GhACS1‐A12* gene was cloned into the pBlue‐script SK vector to create an inverted repeat transgene, which was subsequently inserted into the pBI121 vector. Likewise, ≈250 bp specific sequence of *GhACS1‐D03* was used for constructing the RNAi vector. The constitutive CaMV35S promoter in both pCambia1302 and pBI121 vectors was replaced with the fiber‐specific promoter of *GhRDL1*. All primers were listed in Table S1 (Supporting Information). Cotton genetic transformation was performed with the shoot apical meristem cell‐mediated transformation system.^[^ ^45^ ^]^

### Identification of Ethylene Biosynthesis and Signaling Genes

Genes involved in ethylene biosynthesis and signaling were identified by first compiling a reference set including *MAT/SAM*, *ACS*, *ACO*, *ETR*, *CTR*, *EIN2*, and *EIN3/EIL*, using *Arabidopsis thaliana* (TAIR10) proteins as queries.^[^ ^46^ ^]^ The *G. hirsutum* proteome was searched by BLASTP (E‐value ≤ 1e‐20, coverage ≥ 60%, identity ≥ 30%),^[^ ^47^ ^]^ and orthologs in *G. arboreum* and *G. raimondii* were retrieved. Orthogroups were confirmed by OrthoFinder v2.5.4,^[^ ^48^ ^]^ and homeologous A/D pairs in *G. hirsutum* were verified by synteny analysis with MCScanX.^[^ ^49^ ^]^ Candidate genes were retained only if their domain architectures matched family‐specific profiles.

### RNA Isolation and Quantitative Real‐Time RT‐PCR (qRT‐PCR)

Total RNAs were extracted from 10 to 15 days post anthesis (DPA) fibers of ZM24 (*G. hirsutum*), SXY1 (*G. arboreum*), D_5_‐3 (*G. raimondii*), *PK1* overexpression lines (*G. hirsutum* and *G. arboreum*), *GhACS1‐A12* RNAi and *GhACS1‐D03* RNAi lines (*G. hirsutum*) using the RNA Extraction Kit (Vazyme, Nanjing, China) following the manufacturer's instructions. Briefly, cDNA was synthesized from total RNA and subsequently used as a template in qRT‐PCR with gene specific primers. The PCR was carried out with SYBR‐Green Real‐time PCR Master Mix (Toyobo, Osaka, Japan), and the cotton polyubiquitin gene *GhUBQ7* was used as the reference control, in accordance with a previously described method.^[^ ^23^ ^]^ The mean value and standard deviation of three biological replicates were depicted. All primers were listed in Table S1 (Supporting Information).

### Microscopic Analysis of Cotton Fiber Sections

Cotton fiber morphology was investigated using a variety of techniques. 25, 30 DPA, and mature fiber samples were initially fixed in 4% paraformaldehyde solution and subsequently dehydrated before being embedded in Spurr resin according to the manufacturer's instructions (SPI Supplies, West Chester, PA, USA). Sections of the embedded samples were then obtained using a LEICA EM UC7 ultramicrotome (Leica, Germany) prior to imaging using a Leica SP5 Meta confocal laser microscope (Leica, Germany). At least 50 samples of 10, 15, 20, 25, 30 DPA and mature fibers, each, were evaluated in this study, with measurements taken of both fiber length and cell wall thickness using a ruler and Image J software, respectively.

### Assessing Fiber Quality Characteristics

Mature fiber samples weighing 15 g each were prepared under consistent humidity and temperature conditions prior to testing. The fiber length, strength, and micronaire were measured by Cotton Fiber Quality Inspection and Testing Center of the Chinese Ministry of Agriculture (Anyang, Henan, China) for cotton fiber quality determination.

### Cell Wall Extraction and Cellulose Content Measurement

The pulverized 5, 10, 15, 20, 25, 30 DPA and mature fibers were processed using a high‐throughput tissue grinder, and an aliquot of 0.05 g each sample was mixed with 1 mL of 80% ethanol at room temperature. The mixture was rapidly homogenized. The pellets obtained after centrifugation were rinsed with water at 90 °C for 20 min. Following the removal of the supernatant, crude cell wall materials were obtained by sequentially washing the pellet twice with 80% ethanol and acetone. These materials were subsequently subjected to α‐amylase digestion for 15 h. After centrifugation, the supernatant was discarded and the pellet was washed twice with distilled water before being dried at 90 °C to obtain the cell wall. The content of cellulose in the cotton fiber was determined using a Cellulose Content Assay kit (Solarbio, Beijing, China).

### Ethylene Measurement

Ethylene production was measured in cotton fiber using a gas chromatograph (GC‐2010 Plus; Shimadzu, Japan) equipped with a Flame Ionization Detector (GC‐FID) and 30‐m HP‐PLOT column (RXT‐1), with a sensitivity of 0.01 ppm. Freshly collected 5, 10, 15, 20 DPA fibers of *G. hirsutum* (ZM24) and *PK1* overexpression lines (*G. hirsutum*), as well as 10, 15 DPA fibers of *G. arboreum* (SXY1), *PK1* overexpression lines (*G. arboreum*), *G. raimondii* (D_5_‐3), *GhACS1‐A12‐* and *GhACS1‐D03‐*RNAi plants and corresponding control plants (≈5.0 g each) were placed in 50 mL glass flasks in darkness at 30 °C for 48 h, and the released gas was collected via the drain method using a needle.^[^ ^50^ ^]^ Gas samples (100 µL) were analyzed by GC‐FID. The measurements were recorded for five replicates, and the final assessment results were calculated per gram per hour (nl·g^−1^ FW·h^−1^).

### RNA‐seq Analysis

Total RNAs of 10 and 15 DPA fibers were extracted from ZM24, SXY1, D_5_‐3, *PK1* overexpression, and *GhACS1‐D03* RNAi lines (*G. hirsutum*) using the RNA Extraction Kit (Vazyme, Nanjing, China). The isolated RNAs were used to construct libraries and sequenced on Illumina Novaseq 6000 platform (Biomarker Bioinformatics Technology Co., Ltd, Beijing, China). Three biological repeats were performed for the RNA‐seq experiment. In total, 30 cDNA libraries yielded 191.13 Gb of clean bases; per‐library Q30 values were ≥93.51%. The clean reads were mapped to the *G. hirsutum* (ZM24 genome CRI_v1, https://www.cottongen.org/bio_data/588), *G. arboretum* (SXY1 genome CRI‐updated_v1, https://www.cottongen.org/bio_data/635), and *G. raimondii* (D5 genome JGI_v2_a2.1, https://www.cottongen.org/bio_data/582) reference genome using Hisat2 software StringTie.^[^ ^51^, ^52^ ^]^ Differential gene expression analysis was conducted using the EdgeR, considering genes with a fold change greater than two and a *P* value less than 0.05 as significant.^[^ ^53^ ^]^ The heat map of genes expression was generated based on RNA‐seq data using TBtools software.^[^ ^54^ ^]^ Kyoto Encyclopedia of Genes and Genomes (KEGG) enrichment analysis was performed on differentially expressed genes using the BMKCloud (www.biocloud.net).

### Phylogenetic Analysis

The putative protein sequences of ACS originating from *Arabidopsis thaliana* and *G. hirsutum* were aligned using ClustalX, and a phylogenetic tree was generated via the maximum likelihood method with 1000 bootstrap replications, implementing MEGA7.^[^ ^55^, ^56^ ^]^

### In Vitro Cotton Ovule Culture

Surface‐sterilization of 1 DPA ovules from ZM24, *PK1* overexpression lines (*G. hirsutum*), and *GhACS1‐D03* RNAi lines (*G. hirsutum*) was performed using 75% (v/v) ethanol for 5 min, followed by five washes with sterilized distilled water. The ovules were subsequently immersed in BT liquid medium^[^ ^57^ ^]^ for ovule culture, containing varying concentrations of 1‐aminocyclopropanecarboxylic acid (ACC) and aminoethoxyvinylglycine (AVG), and incubated in darkness at a constant temperature of 28 ± 1 °C for a period of 10 days. The culture medium was refreshed every two days to maintain optimal conditions.

### Yeast Two‐Hybrid (Y2H) Assay

To investigate protein‐protein interactions in cotton, the yeast two‐hybrid system was employed using the Matchmaker Gold Yeast Two‐Hybrid System (Clontech, USA). The coding sequences of the *PK1*, *AtACS5*, and *GhACS*s were amplified and cloned into pGBKT7 and pGADT7 vectors, respectively. The transformation of both vectors was performed in the yeast strain Y2HGold using a standard lithium acetate method.^[^ ^58^ ^]^ The transformed cells were subjected to selection on SD/‐Leu/‐Trp medium, followed by subsequent screening on higher stringency medium SD/‐Ade/‐His/‐Leu/‐Trp with X‐α‐Gal and Aureobasidin A. All primers were listed in Table S1 (Supporting Information).

### Luciferase Complementation Imaging (LCI) Assay

The pCAMBIA1300‐cLUC vector was utilized to clone the full‐length coding sequence of *PK1*, while the pCAMBIA1300‐nLUC vector was used for cloning *GhACS1‐A12* and *GhACS1‐D03*, and subsequently transformed into the GV3101‐pSoup‐P19 Agrobacterium strain. The bacterial suspension (OD600 = 1.0) was collected through centrifugation and resuspended in a mixture of 50 mmol L^−1^ MES (pH 5.7), 2 mmol L^−1^ Na_3_PO_4_•12H_2_O, and 150 µmol L^−1^ acetosyringone combined with 500 mg/100 mL D‐glucose. After a 2–3 h incubation at room temperature, the two bacterial solutions were mixed in a 1:1 ratio and injected into *Nicotiana benthamiana* leaves, after which fluorescence signals were observed using a multifunctional imager (Tanon, 5200 Multi, China). All primers were listed in Table S1 (Supporting Information).

### Co‐Immunoprecipitation (Co‐IP) Assay

To conduct a Co‐IP assay, the coding sequences of *PK1*, *GhACS1‐A12*, and *GhACS1‐D03* were amplified without the stop codon and fused with the GFP and Flag tags, respectively. Subsequently, these vectors were transformed into the GV3101‐pSoup‐P19 Agrobacterium strain. Four‐week‐old tobacco leaves were infiltrated with the resulting recombinant proteins and then incubated in darkness for 24 h, followed by 24 h of light exposure. The proteins were extracted using a buffer solution comprising 50 mm Tris–HCl, pH 7.5, 1 mm EDTA, 150 mm NaCl, 20% glycerol, 2% Triton‐X‐100, 20 µg mL^−1^ of MG‐132 (Sigma–Aldrich, USA), and 1 × protease inhibitor cocktail (Roche, Germany). Immunoprecipitation was carried out with protein A/G plus agarose (Santa Cruz Biotechnology, USA) after co‐incubating the extracts with this agarose to minimize the nonspecific binding. The addition of anti‐GFP antibody (Abmart, China) was followed by 1 h of incubation at 4 °C. The samples were then precipitated, washed at least four times, eluted with 1 × SDS protein loading buffer, and boiled for 3 min. The proteins were finally detected using anti‐GFP antibody and anti‐Flag antibody (Abmart, China). All primers were listed in Table S1 (Supporting Information).

### Yeast One‐Hybrid (Y1H) Assay

Yeast one‐hybrid assay was performed using the Matchmaker Gold Yeast One‐Hybrid System (Clontech, USA). Promoter fragments of potential target genes for EIN3/EIL and ERF were cloned into pAbAi vectors as bait constructs. The plasmids were linearized and transformed into the Y1H Gold strain to generate bait‐specific reporter strains. Each bait‐specific reporter yeast strain was subsequently transformed with the pGADT7 vector as a negative control. Protein‐DNA interactions were assessed based on the growth ability of the co‐transformants on SD/‐Leu medium supplemented with Aureobasidin A (AbA), following the manufacturer's protocol. All primers were listed in Table S1 (Supporting Information).

### Dual‐Luciferase Reporter Assay

In the dual‐luciferase reporter assay, the effector plasmid pGreen II 62‐SK was modified to include the full‐length coding sequences of *GhEIN3‐D08* and *GhERF‐A11*. In addition, the reporter plasmid pGreen II 0800‐LUC was constructed by cloning 2000 bp promoter sequences of potential target genes. The transformed plasmids were introduced into the Agrobacterium strain GV3101 and co‐infiltrated into *N. benthamiana* leaves. Following a three‐day incubation after infiltration, the bioluminescence intensity of the firefly luciferase was observed using an imaging system (Tanon, 5200 Multi, China). Firefly luciferase and renilla luciferase activities within the *N. benthamiana* leaves were measured using a Dual‐Luciferase reporter kit (Promega, USA) according to the manufacturer's instructions. All primers were listed in Table S1 (Supporting Information).

### Electrophoretic Mobility Shift Assay (EMSA)

The coding sequences of *GhEIN3‐D08* and *GhERF‐A11* were cloned into pET28a vector and expressed in the *E. coli* strain BL21. The recombinant His‐GhEIN3‐D08 and His‐GhERF‐A11 proteins were purified using a His‐tagged Fusion Protein Purification kit (ThermoFisher Scientific, USA) as per the manufacturer's instructions. The DNA fragments were then incubated with 300 ng His‐GhEIN3‐D08 in binding buffer (750 mm KCl, 0.5 mm dithiothreitol, 0.5 mm EDTA, 50 mm Tris, pH 7.4) for 20 min. The protein‐bound DNA fragments were separated from the unbound ones by non‐denaturing polyacrylamide gel electrophoresis (PAGE). For competition analysis, promoter fragments were FAM‐labeled at the 5′‐end. Unlabeled promoter fragments were included in the reactions as competitors in a 10‐ and 100‐fold molar excess relative to the labeled probes. Fluorescence was visualized using an imaging system (Tanon, 5200 Multi, China). All primers were listed in Table S1 (Supporting Information).

### Semi‐In Vivo Protein Degradation Assay

To conduct a semi‐in vivo protein degradation assay, 10 DPA ovules from both ZM24 and *PK1* overexpression lines (*G. hirsutum*) were first surface‐sterilized. These ovules were then immersed in BT liquid medium containing DMSO and 50 µm MG132 and incubated in darkness at a constant temperature of 28 ± 1 °C for 18 h. Following this incubation, fiber samples were collected, and the proteins were extracted using a buffer solution containing 50 mm Tris‐HCl, pH 8.0, 10 mm EDTA, 1 mm MgCl_2_, 0.5 m sucrose, 5 mm DTT, and 1 × protease inhibitor cocktail. To analyze the protein levels, immunoblotting experiments were conducted using self‐prepared rabbit polyclonal anti‐GhACS1‐A12 and anti‐GhACS1‐D03 antibodies, with β‐actin antibody serving as a control.

### In Vivo Ubiquitination Assay

To examine the ubiquitination levels of GhACS1‐A12 and GhACS1‐D03, 10 DPA fibers from ZM24 and *PK1* OE lines were harvested and ground in liquid nitrogen. Total proteins were extracted with a buffer containing 50 mm Tris‐HCl (pH 7.5), 150 mm NaCl, 1 mm EDTA, 20% glycerol, 2% Triton X‐100, 20 µg mL^−1^ MG‐132 (Sigma–Aldrich, USA), 10 mm N‐ethylmaleimide (NEM; Sigma–Aldrich, USA), and 1 × protease inhibitor cocktail (Roche, Germany). After centrifugation at 16 000 × g for 15 min at 4 °C, the supernatants were collected and pre‐incubated with protein A/G plus agarose (Santa Cruz Biotechnology, USA) for 1 h to reduce nonspecific binding. The extracts were then immunoprecipitated using anti‐GhACS1‐A12 or anti‐GhACS1‐D03 antibodies at 4 °C for 2 h, followed by incubation with protein A/G plus agarose for 1 h. The beads were washed at least four times with extraction buffer, and bound proteins were eluted by boiling in 1 × SDS loading buffer for 3 min. Ubiquitination levels were detected by immunoblotting using anti‐ubiquitin antibody (Santa Cruz Biotechnology, USA).

### Statistical Analysis

All experiments were performed with at least three independent biological replicates. Data were processed using Microsoft Excel, and statistical significance was determined using a two‐tailed Student's *t*‐test. Asterisks denote significance levels: ^*^, *P* < 0.05; ^**^, *P* < 0.01.

## Conflict of Interest

The authors declare no conflict of interest.

## Author Contributions

Z.R.Y. conceived and designed the research. J.Z., M.Y., J.L., and G.G. performed the experiments. J.Z., Y.G., and G.H. analyzed data. S.M. provided the transgenic materials. J.Z. prepared the draft of the manuscript. J.Z. and Z.E.Y. wrote the paper. All authors read and approved the final manuscript.

## Supporting information



Supporting Information

Supporting Information

## Data Availability

RNA‐seq data have been deposited in NCBI under BioProject PRJNA[1309839].
