# Supplementary material for: Evolutionary Divergence of an Ethylene‐Responsive Transcriptional Cascade Governs a Dose‐Dependent Balance between Cotton Fiber Length and Strength
Source: Adv Sci (Weinh). 2025 Nov 7;13(5):e14154. doi: 10.1002/advs.202514154 (PMC12850088; doi:10.1002/advs.202514154)
Supplement: Supplementary file 1 — Supporting Information [file ADVS-13-e14154-s001.docx]

**Figures S1-19 and Table S6, Supporting Information**


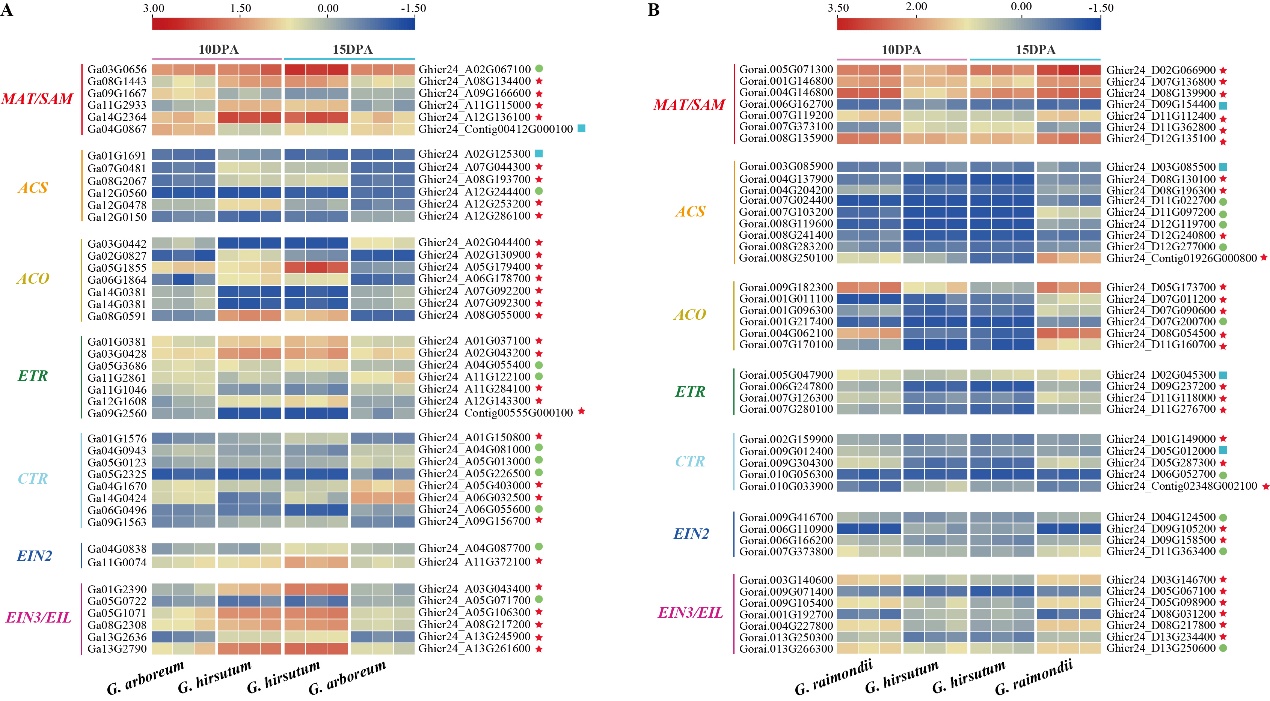


**Figures S1**. Classification and transcript levels of ethylene-related differentially expressed genes (DEGs) in *Gossypium hirsutum*, *Gossypium arboreum* and *Gossypium* *raimondii* fibers. (A) Classification and transcript levels of DEGs related to ethylene biosynthesis and signal transduction in 10- and 15 DPA fibers of *G. hirsutum* and *G. arboreum*. The gene IDs on the left correspond to *G. arboreum*, while those on the right correspond to *G. hirsutum*. (B) Classification and transcript levels of DEGs related to ethylene biosynthesis and signal transduction in 10 and 15 DPA fibers of *G. hirsutum* and *G.* *raimondii*. The gene IDs on the left correspond to *G.* *raimondii*, while those on the right correspond to *G. hirsutum*. The relative expression of each gene was calculated using the log_2_(FPKM) formula. Blue squares indicate genes that are differentially expressed in 10 DPA fibers between *G. hirsutum* and *G. arboreum*, or between *G. hirsutum* and *G.* *raimondii*. Green circles indicate genes that are differentially expressed in 15 DPA fibers between *G. hirsutum* and *G. arboreum*, or between *G. hirsutum* and *G.* *raimondii*. Red asterisks indicate genes that are differentially expressed in both 10 and 15 DPA fibers between *G. hirsutum* and *G. arboreum*, or between *G. hirsutum* and *G.* *raimondii*.


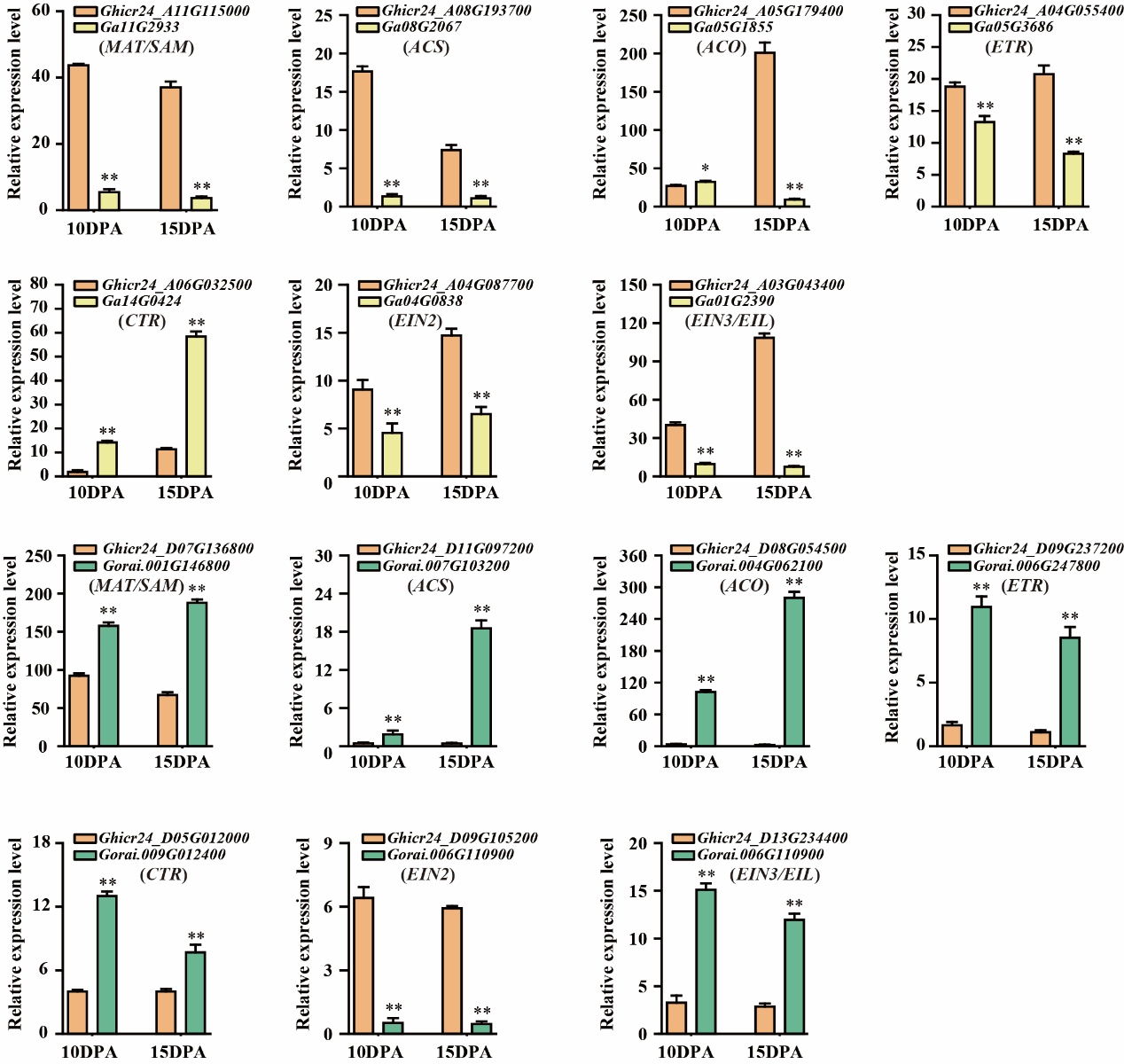


**Figures S2**. Expression levels of several genes related to ethylene in 10 and 15 DPA fibers from *G. hirsutum*, *G. arboreum* and *G.* *raimondii*. The presented data are the mean values and standard deviations from three independent biological replicates. Student’s *t*-tests were conducted and revealed significant differences (*, *P* < 0.05; **, *P* < 0.01) in gene expression levels between *G. hirsutum* and *G. arboreum*, or between *G. hirsutum* and *G.* *raimondii*.


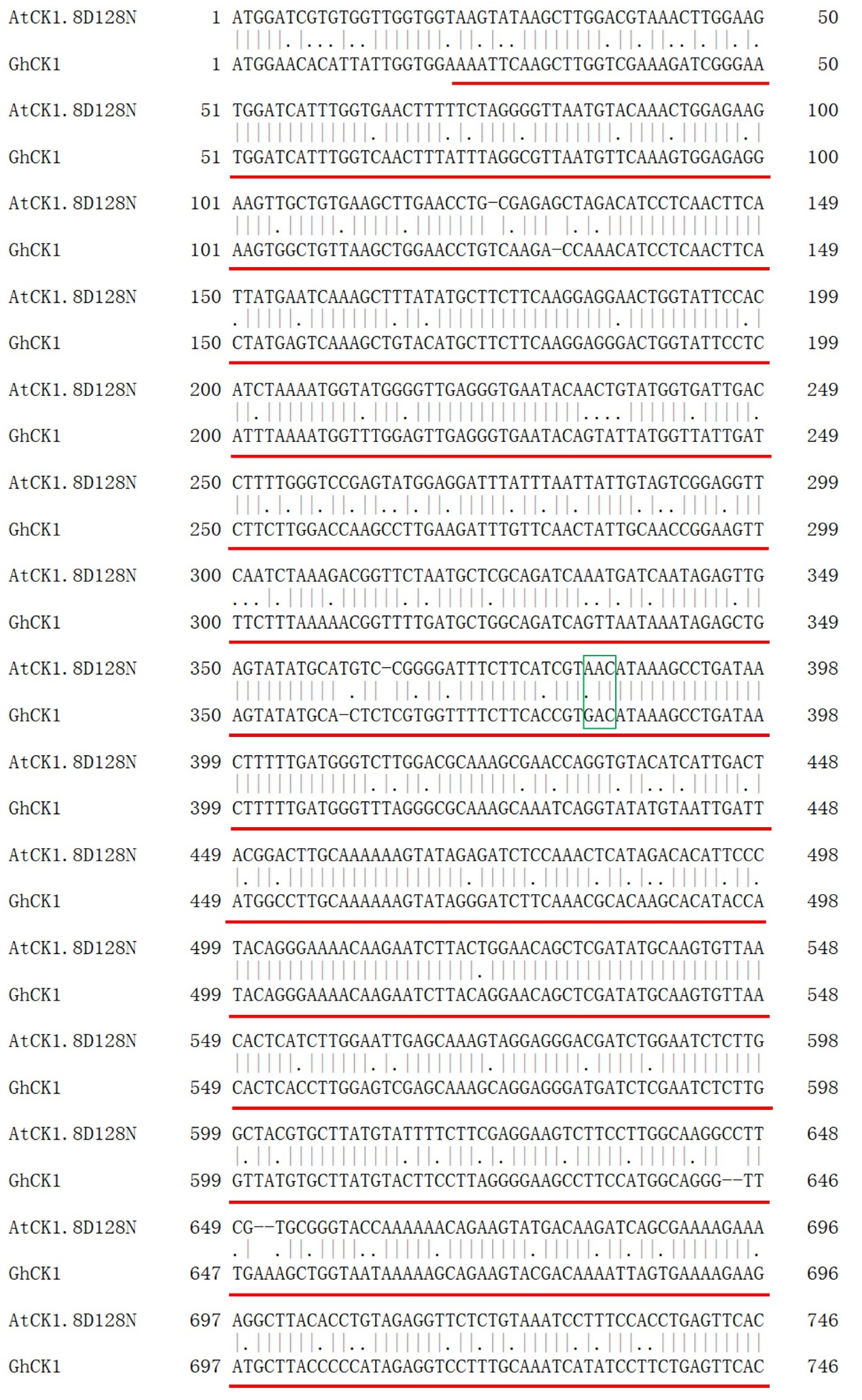


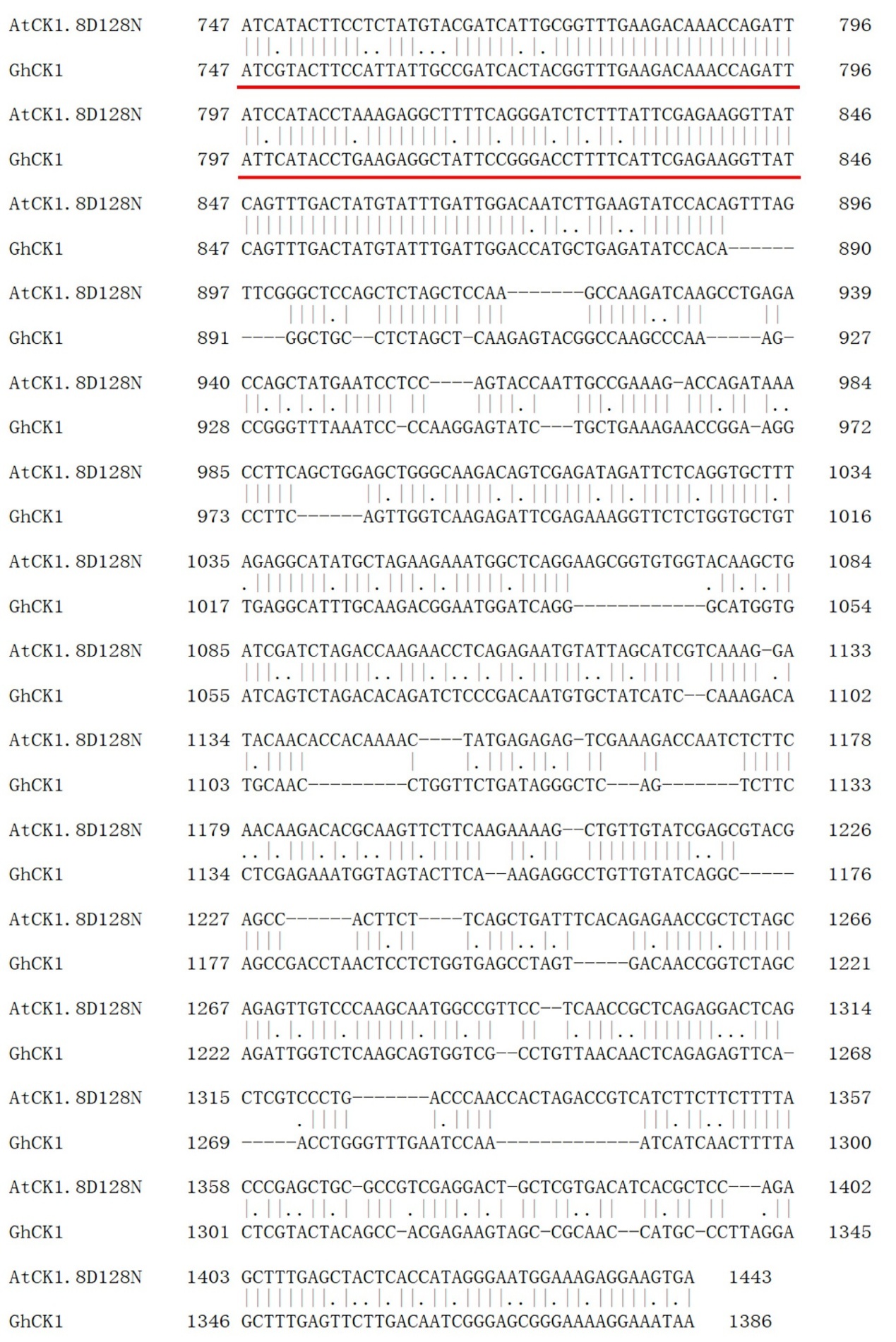


**Figure S3**. Sequence alignment of *AtCK1.8^D128N^* and *GhCK1*.Pairwise nucleotide sequence alignment between *Arabidopsis thaliana* *CK1.8^D128N^* (*AtCK1.8^D128N^*) and *Gossypium hirsutum* *CK1* (*GhCK1*). Red lines indicate the conserved kinase domain regions. The green box marks the mutation site (Asp128 to Asn) in AtCK1.8.


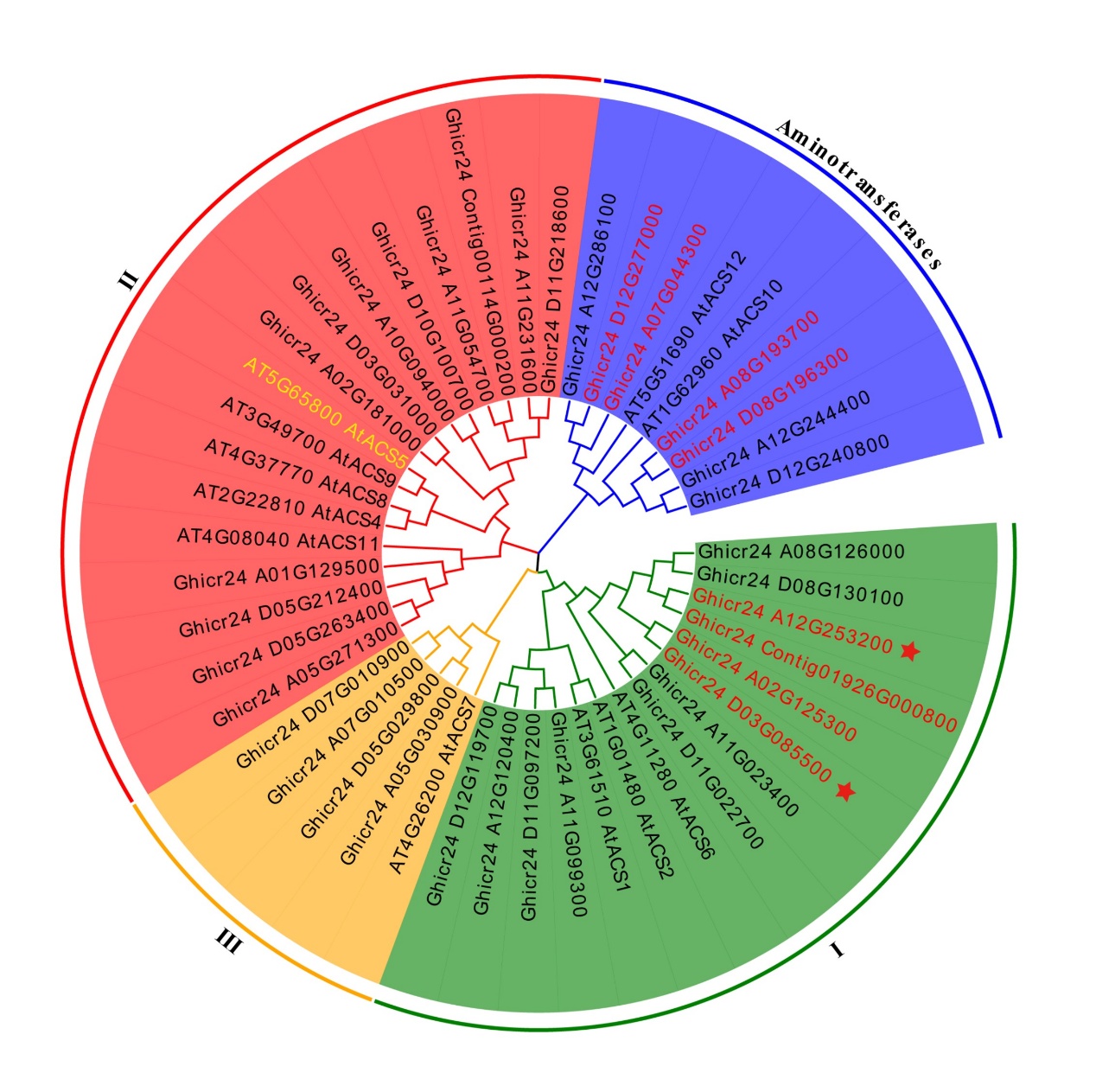


**Figures S4**. Phylogenetic tree of ACS proteins from *Arabidopsis thaliana* and *G. hirsutum*. The protein sequences were aligned using ClustalX and the phylogenetic tree was constructed using the maximum likelihood method with 1000 bootstrap replications in MEGA7. The different types of ACS proteins are shown in different colors, with AtACS5 highlighted in yellow, eight cotton ACS proteins exhibiting high expression in fiber highlighted in red, and two ACS proteins, which interact with PK1, marked with asterisks.


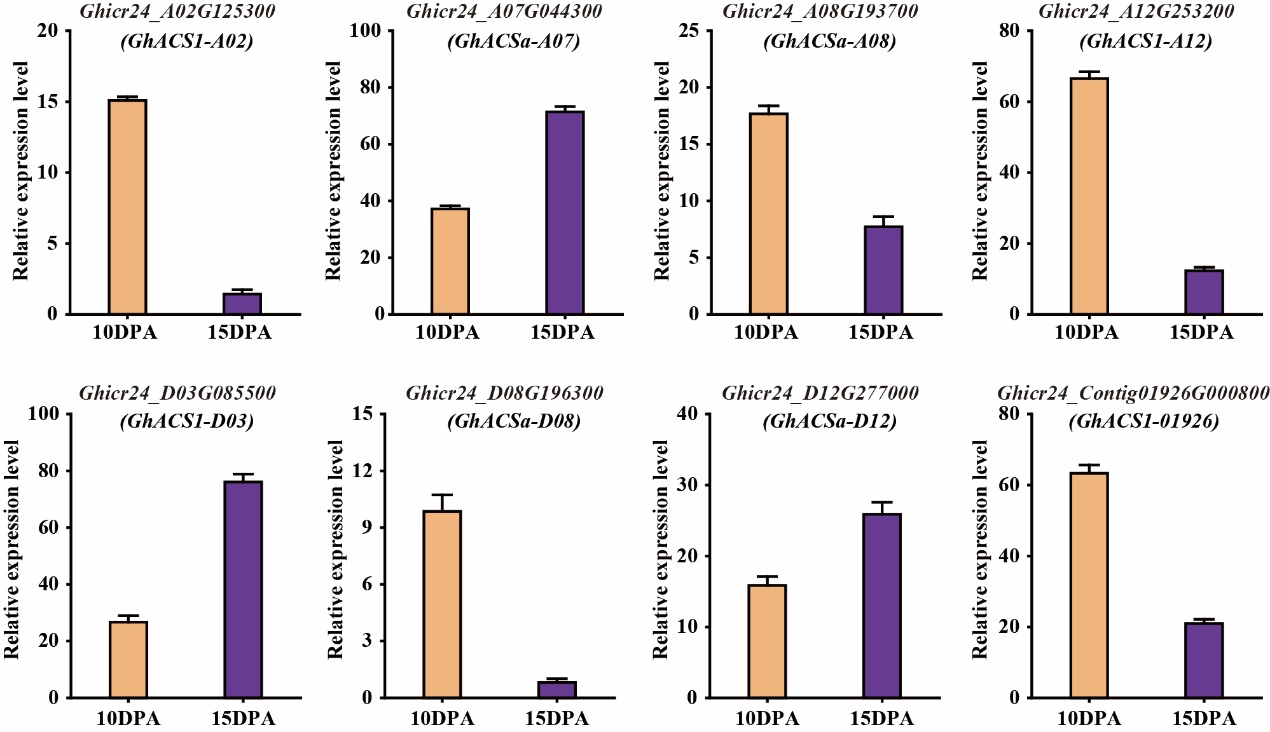


**Figures S5**. Expression profiling of eight cotton *ACS* genes in 10 and 15 DPA fibers from *G. hirsutum*. The relative value of gene expression is presented as a percentage of *GhUBQ7* expression activity. The data shown represent the mean values and standard deviations obtained from three independent biological replicates.


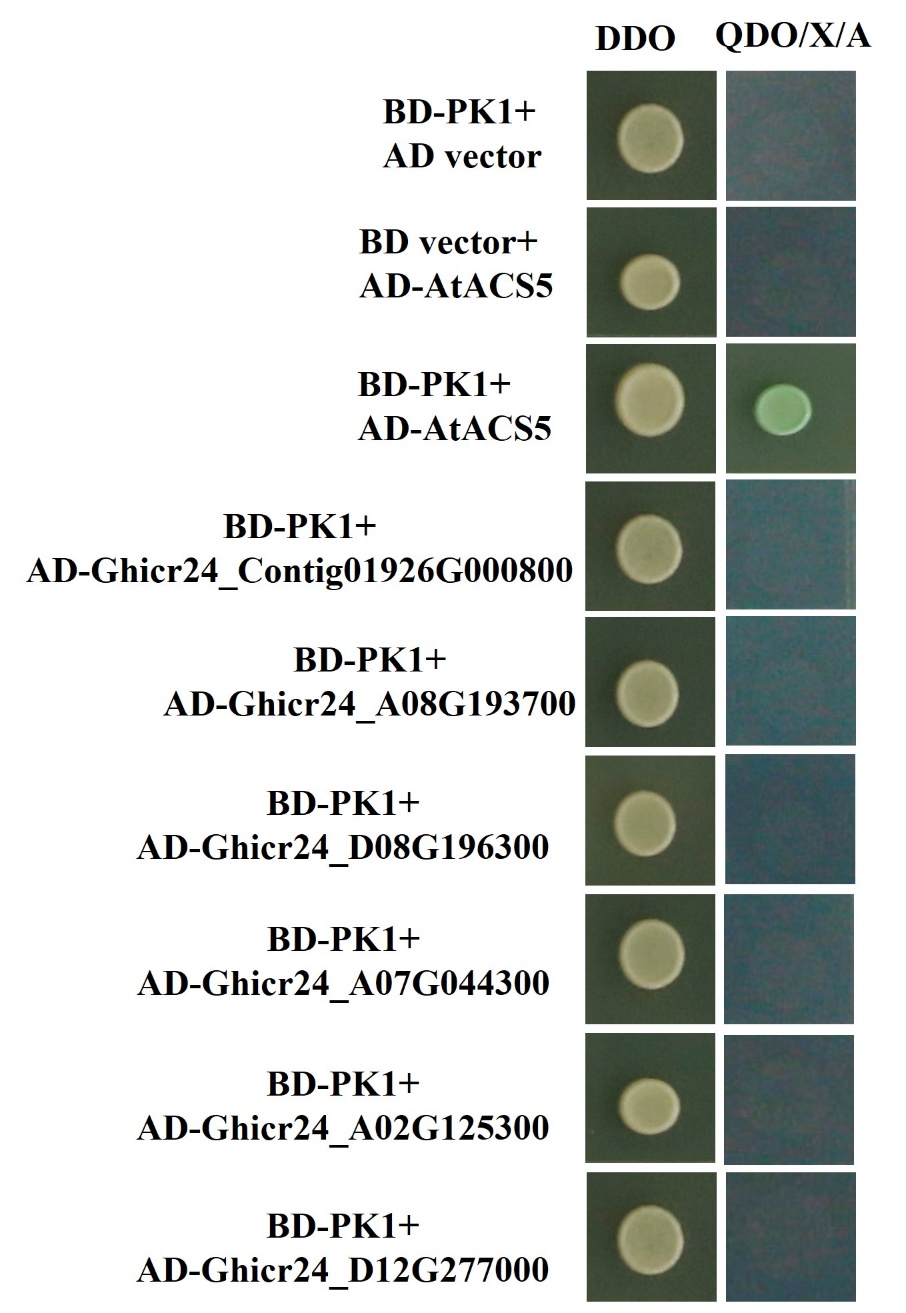


**Figures S6**. Yeast two-hybrid assay to investigate the interactions between PK1 and Ghicr24_Contig01926G000800, Ghicr24_A08G193700, Ghicr24_D08G196300, Ghicr24_A07G044300, Ghicr24_A02G125300 and Ghicr24_D12G277000. Yeast transformants were selected on DDO medium (SD/-Leu/-Trp medium) and the higher stringency QDO/X/A medium (SD/-Ade/-His/-Leu/-Trp with X-α-Gal and Aureobasidin A). The negative controls were BD-PK1+AD vector and BD vector+AD-AtACS5, while BD-PK1+AD-AtACS5 was used as a positive control.


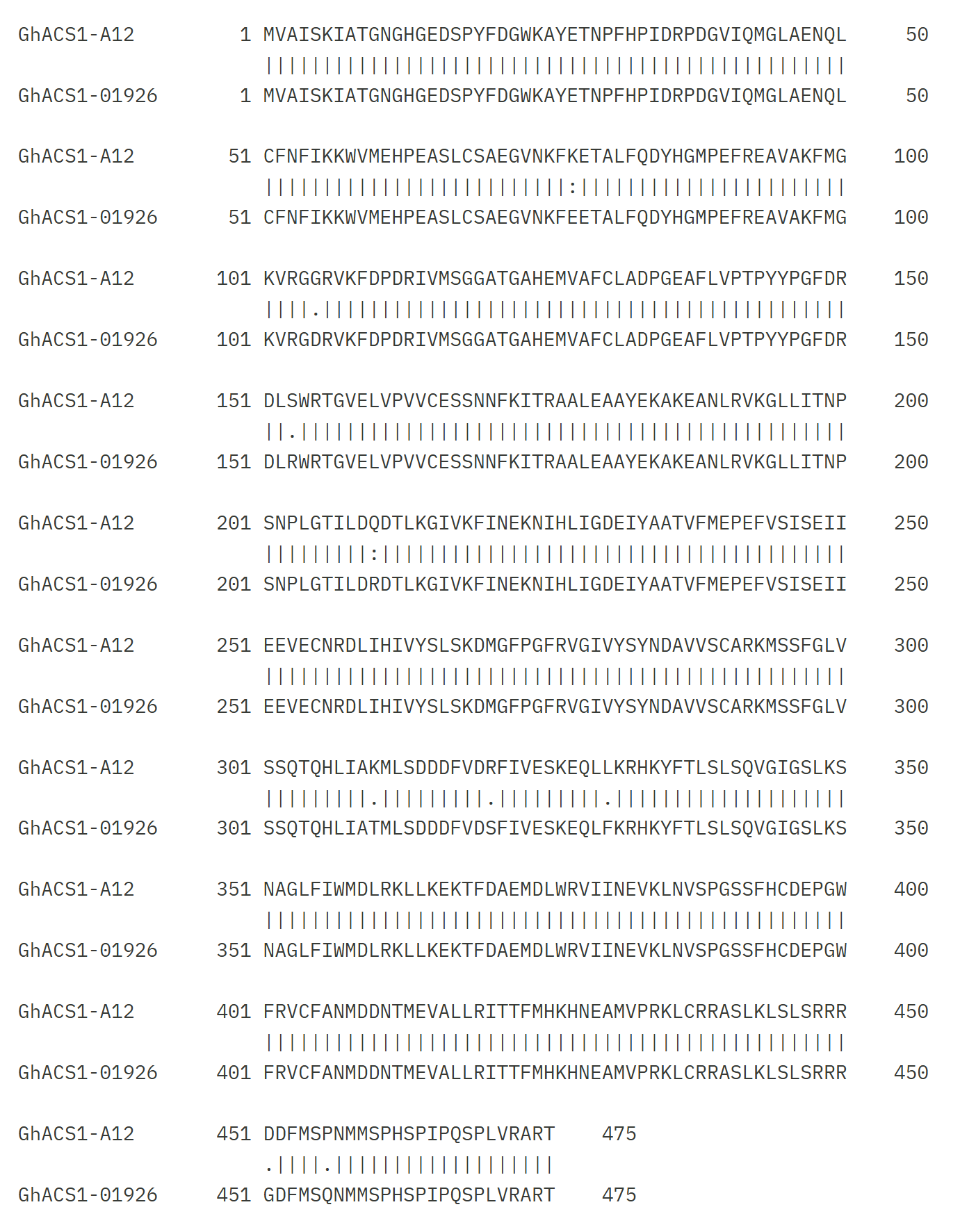

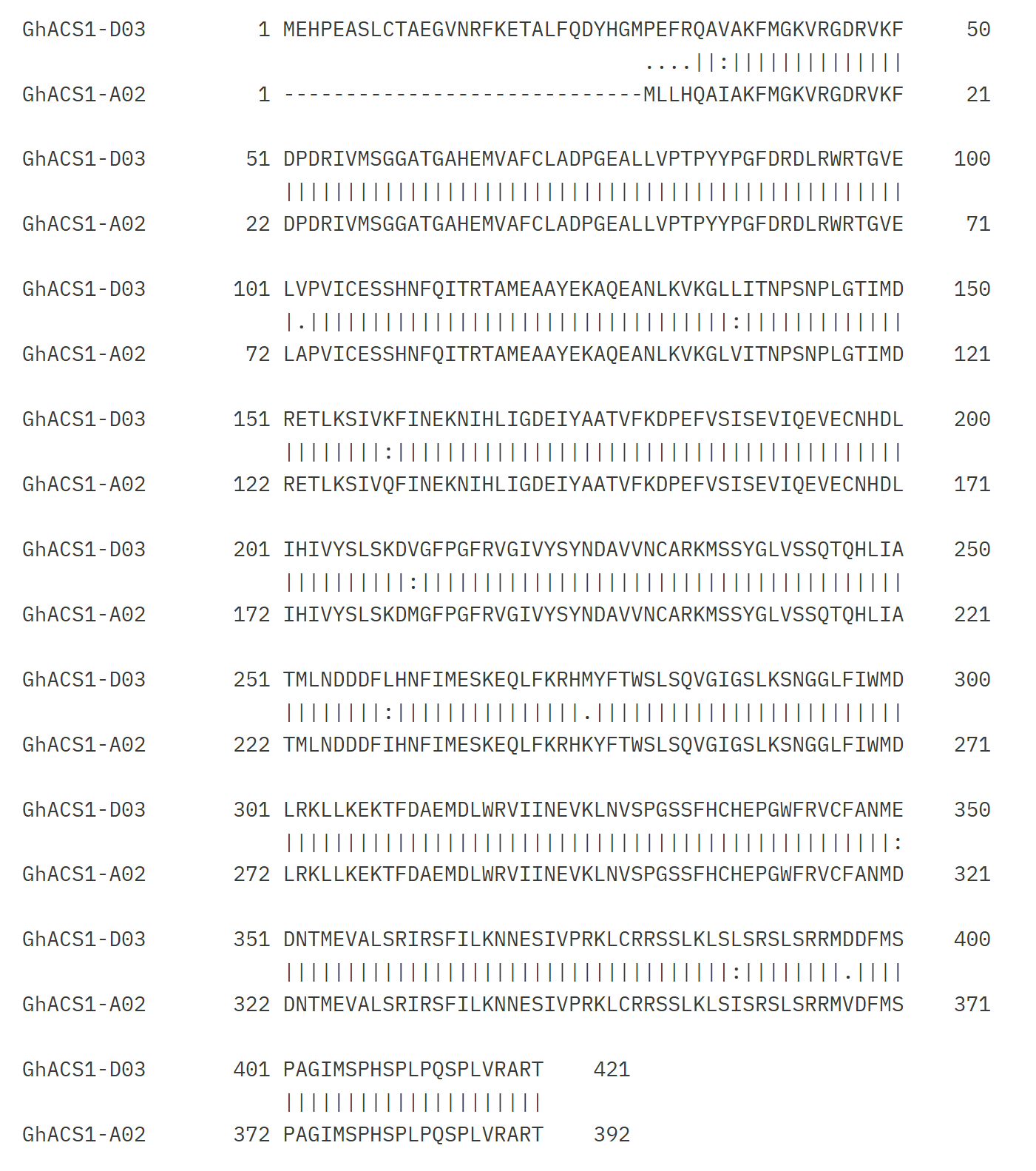


**Figures S7**. Divergence of the C‑terminal TOE‑like motif between PK1‑binding ACS1 isoforms and their direct homoeologs. Pairwise alignments of GhACS1‑A12 with its D‑subgenome homoeolog GhACS1‑01926 and of GhACS1‑D03 with its A‑subgenome homoeolog GhACS1‑A02 highlight a TOE‑like region near the C terminus (red boxes) comprising an RLS‑like segment followed by an acidic patch.


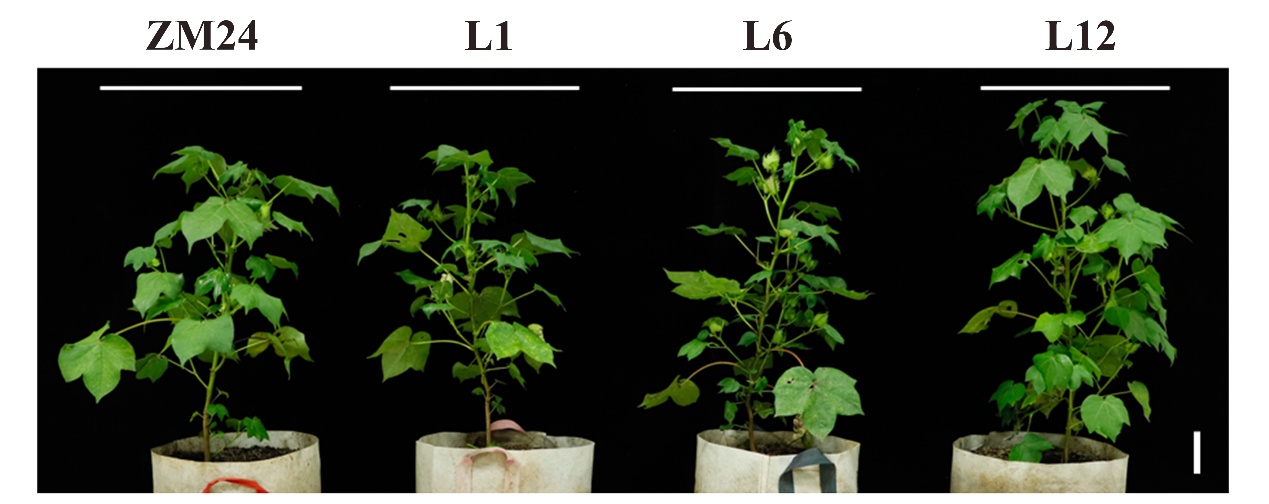


**Figures S8**. The ZM24 and *PK1* overexpression lines growing in soil. Bar = 5 cm. ZM24 is the wild-type plant, while L1, L6 and L12 denote three independent lines of *PK1* overexpression transgenic cotton (*G. hirsutum*).


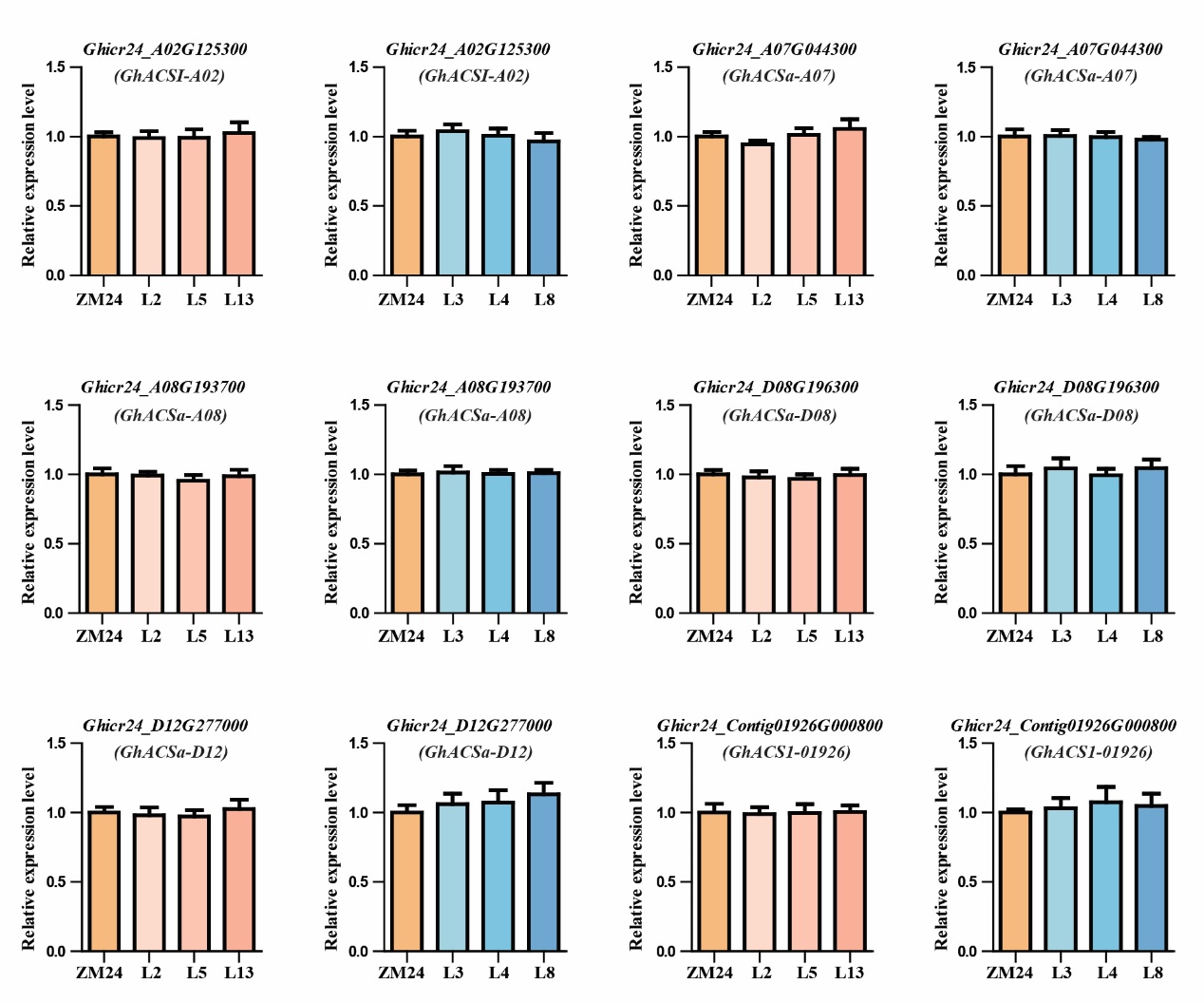


**Figures S9**. Expression levels of non-target, fiber-enriched *ACS* genes in 10 DPA fibers form ZM24, *GhACS1-A12* RNAi and *GhACS1-D03* RNAi lines. The presented data are the mean values and standard deviations from three independent biological replicates. Student’s *t*-tests were conducted and revealed no significant difference (*P* > 0.05) in gene expression levels between ZM24 and *GhACS1-A12* RNAi lines as well as between ZM24 and *GhACS1-D03* RNAi lines. ZM24 is the WT, while L2, L5 and L13 are *GhACS1-A12* RNAi lines and L3, L4 and L8 are *GhACS1-D03* RNAi lines.


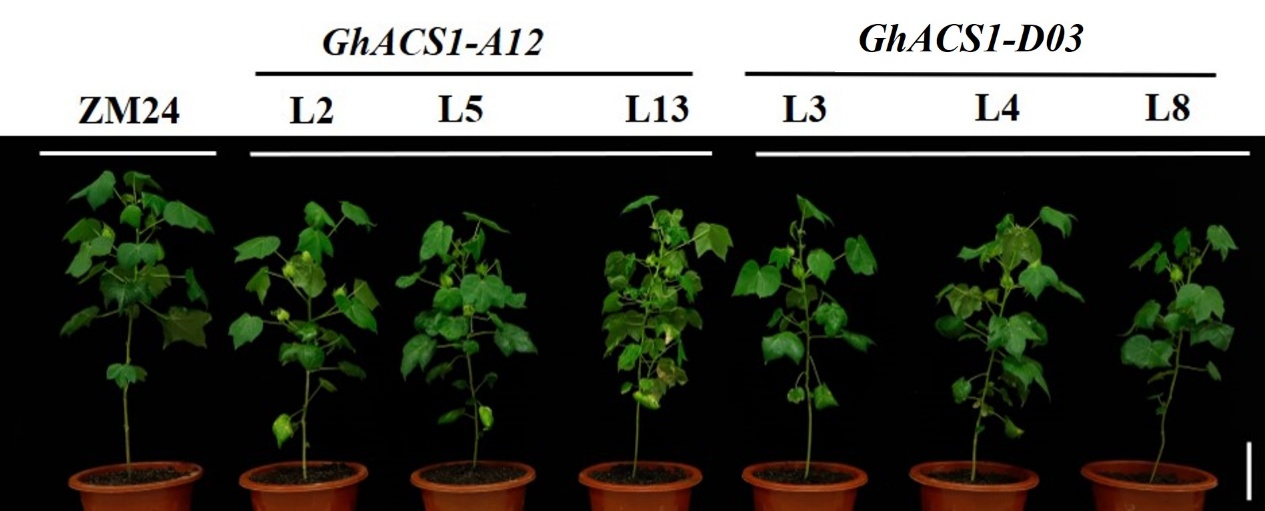


**Figures S10**. The plant architecture of ZM24 as well as *GhACS1-A12* and *GhACS1-D03* RNA interference (RNAi) plants. Bar = 10 cm.


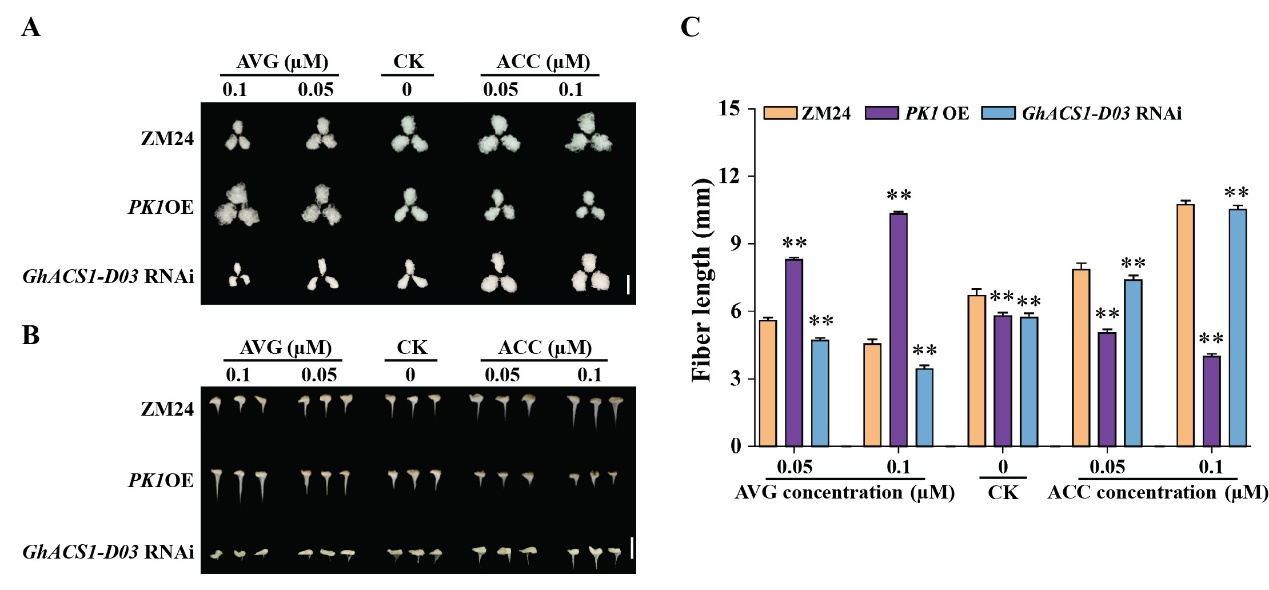


**Figures S11**. Effects of 1-aminocyclopropanecarboxylic acid (ACC) and aminoethoxyvinylglycine (AVG) on fiber length of ZM24, *PK1* overexpression (OE) and *GhACS1-D03* RNAi cotton ovules. (A) The phenotypes of ZM24, *PK1* OE and *GhACS1-D03* RNAi cotton ovules cultured in medium with or without the treatment of ACC and AVG. Bar = 1 cm. (B) Comparison of fiber lengths among ZM24, *PK1* OE and *GhACS1-D03* RNAi cotton ovules cultured in medium with or without ACC and AVG treatment. Bar = 1 cm. (C) Statistical analysis of fiber length measurements for ZM24, *PK1* OE and *GhACS1-D03* RNAi cotton ovules cultured in medium with or without ACC and AVG treatment (n > 30). The mean values and standard deviations are presented based on three biological replicates. The independent *t*-tests indicated significant differences (*, *P* < 0.05; **, *P* < 0.01) between the fiber lengths of ZM24 and *PK1* OE cotton as well as ZM24 and *GhACS1-D03* RNAi cotton. CK represents ovules cultured without ACC and AVG treatment.


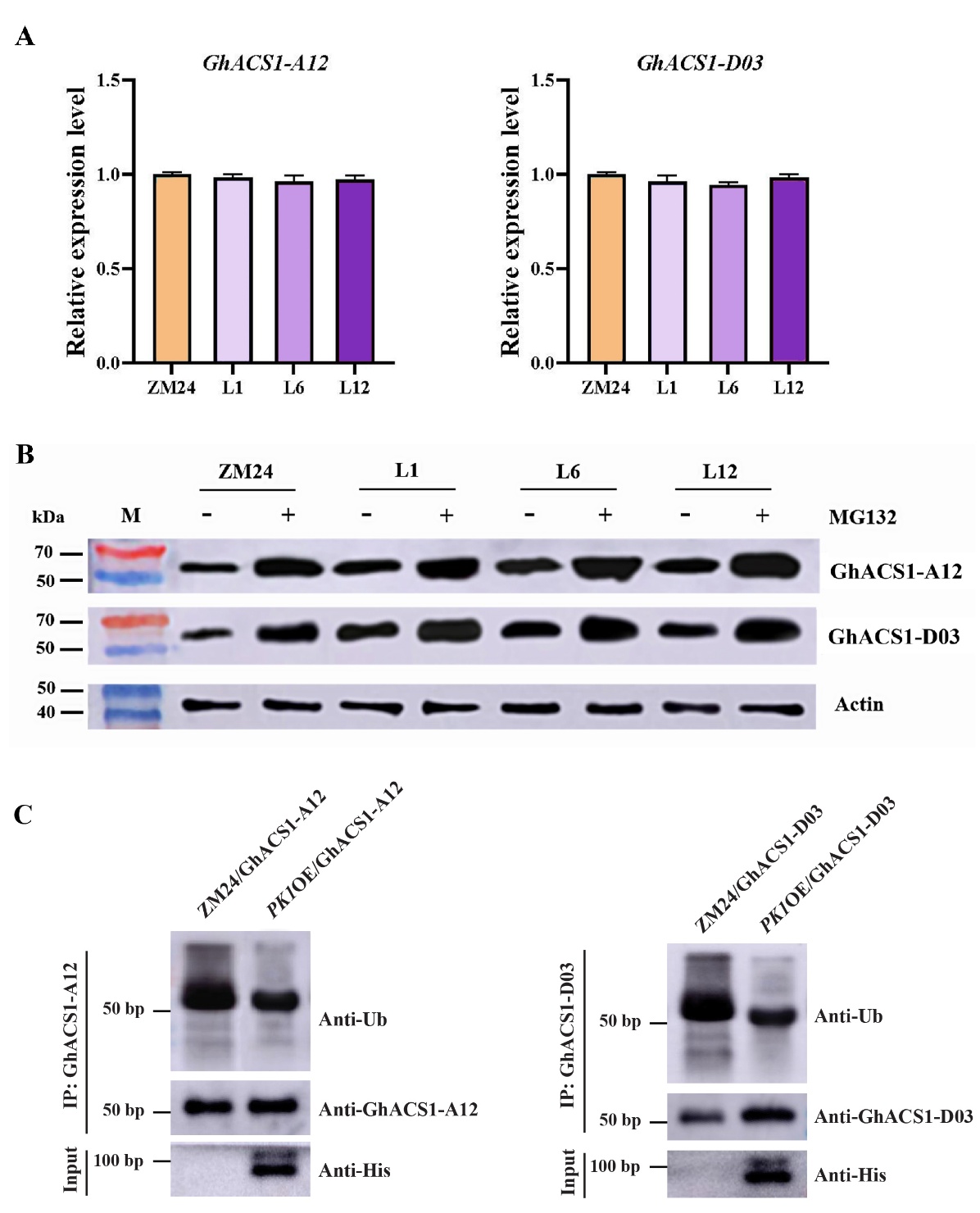


**Figures S12**. PK1 enhances the protein stability of GhACS1 isoforms without affecting their transcription. (A) Expression levels of *GhACS1-A12* and *GhACS1-D03* in 10 DPA fibers from ZM24 and *PK1* overexpression (OE) lines. The presented data are the mean values and standard deviations from three independent biological replicates. Student’s *t*-tests revealed no significant differences (*P* > 0.05) in gene expression levels between ZM24 and *PK1* overexpression lines. (B) Immunoblotting analysis of GhACS1-A12 and GhACS1-D03 protein levels in 10 DPA cotton fibers from ZM24 and *PK1* overexpression lines with or without the proteasome inhibitor MG132 treatment. (C) *In vivo* ubiquitination of GhACS1-A12 and GhACS1-D03 in 10 DPA fibers from ZM24 and *PK1* OE lines. Proteins were immunoprecipitated with anti-GhACS1-A12 and anti-GhACS1-D03, and immunoblotted with anti-ubiquitin.


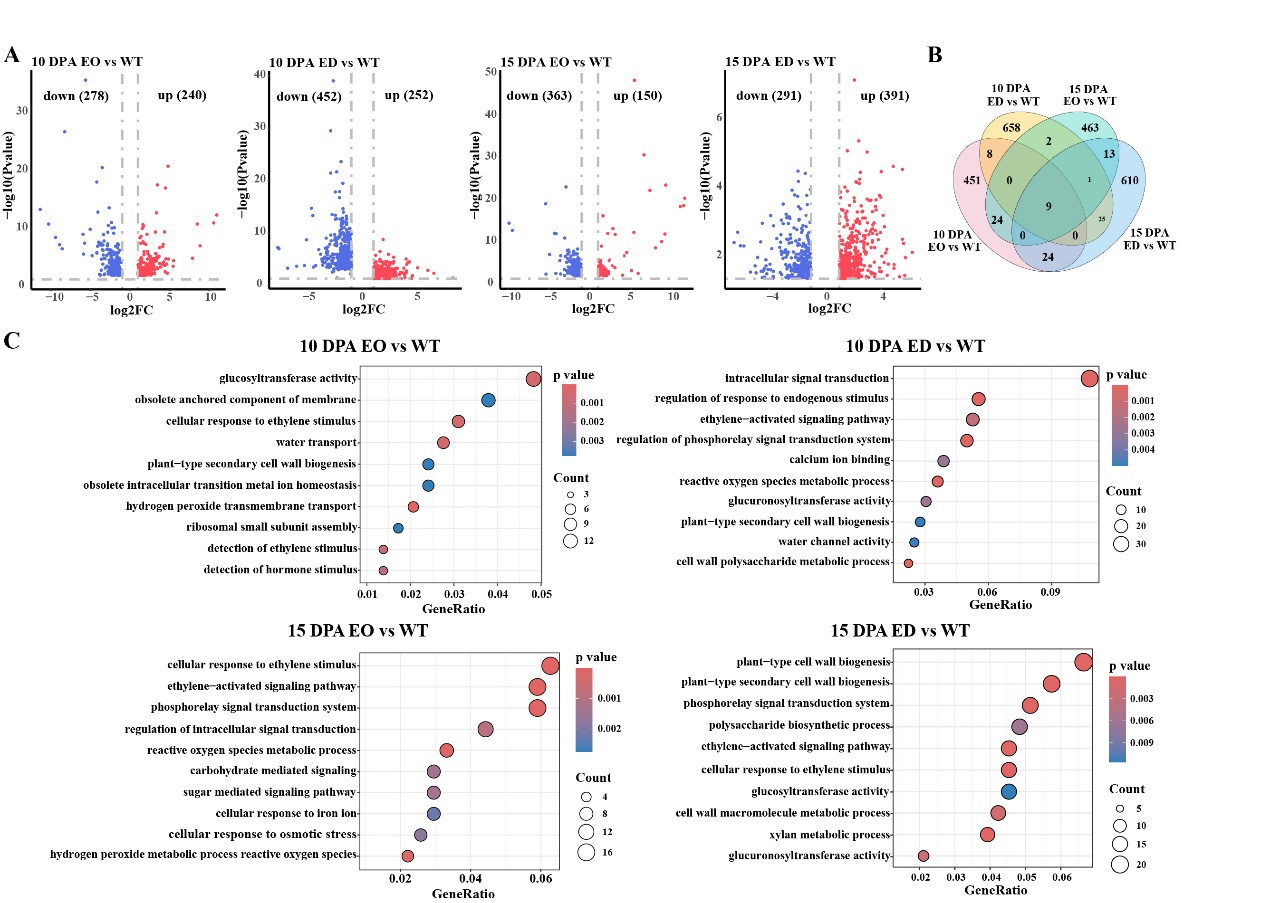


**Figures S13**. Global transcriptome changes between the ethylene overproduction (EO)/ethylene deficient (ED) lines and WT at 10 and 15 DPA. (A) Volcano plots summarizing differentially expressed genes (DEGs) identified in four pairwise comparisons: 10 DPA-EO vs WT, 10 DPA-ED vs WT, 15 DPA-EO vs WT and 15 DPA-ED vs WT. The horizontal dashed line denotes the significance threshold (adjusted *P* < 0.05); vertical dashed lines indicate the log_2_ fold change (log_2_FC) cut-off of ±1. Genes significantly up-regulated (log_2_FC ≥ 1) are displayed in red, down-regulated genes (log_2_FC ≤ –1) in blue. The number of up- and down-regulated transcripts is indicated in parentheses. (B) Venn diagram showing the overlap of DEGs among the four comparisons. Numbers in each sector represent genes that are uniquely or commonly regulated in the indicated datasets. (C) Gene Ontology (GO) enrichment of DEGs. The top 10 significantly enriched GO terms (adjusted *P* < 0.05) are plotted for each comparison. Dot size corresponds to the number of DEGs assigned to the term (Count), whereas colour reflects the adjusted *P*-value. GeneRatio denotes the proportion of DEGs annotated to the term relative to the total number of DEGs in that comparison.


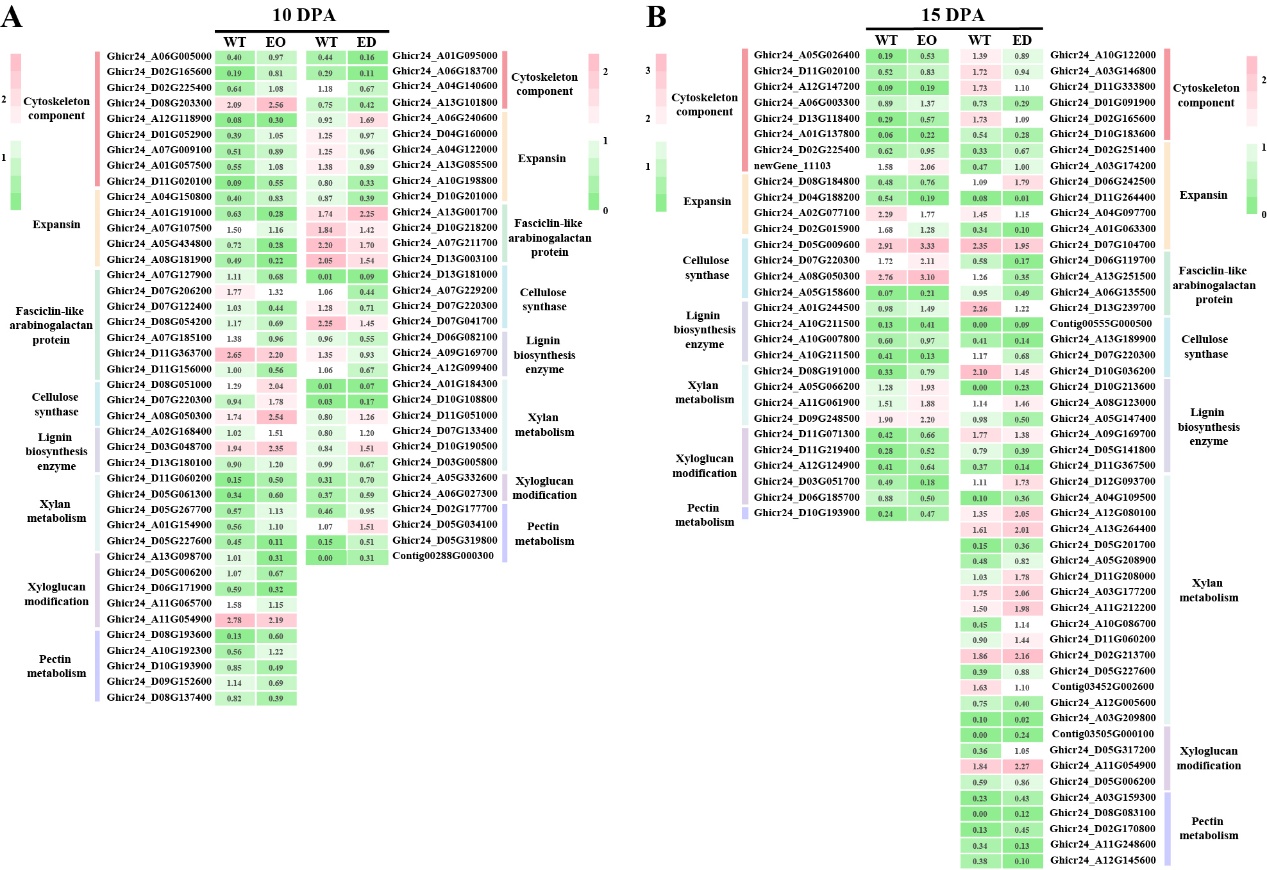


**Figures S14**. Heat‑maps of fiber developmental genes in ethylene overproduction (EO) and ethylene deficient (ED) plants at 10 DPA (A) and 15 DPA (B).


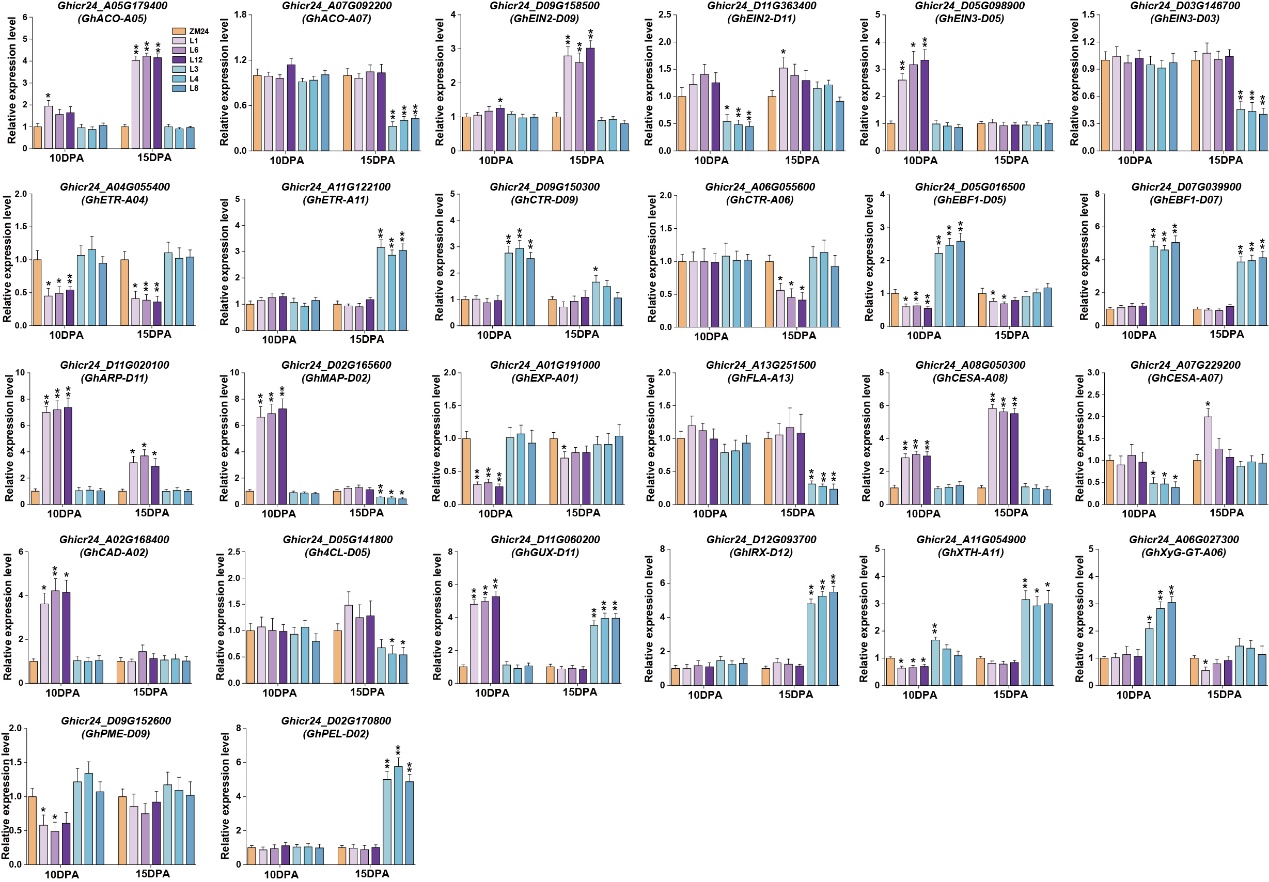


**Figures S15**. Expression levels of several genes related to ethylene biosynthesis and signaling, and fiber development in 10 and 15 DPA fibers from WT and ethylene overproduction (EO)/ethylene deficient (ED) lines. The presented data are the mean values and standard deviations from three independent biological replicates. Student’s *t*-tests were conducted and revealed significant differences (*, *P* < 0.05; **, *P* < 0.01) in gene expression levels between WT and EO lines as well as between WT and ED lines. ZM24 is the WT, while L1, L6 and L12 are EO lines and L3, L4 and L8 are ED lines.


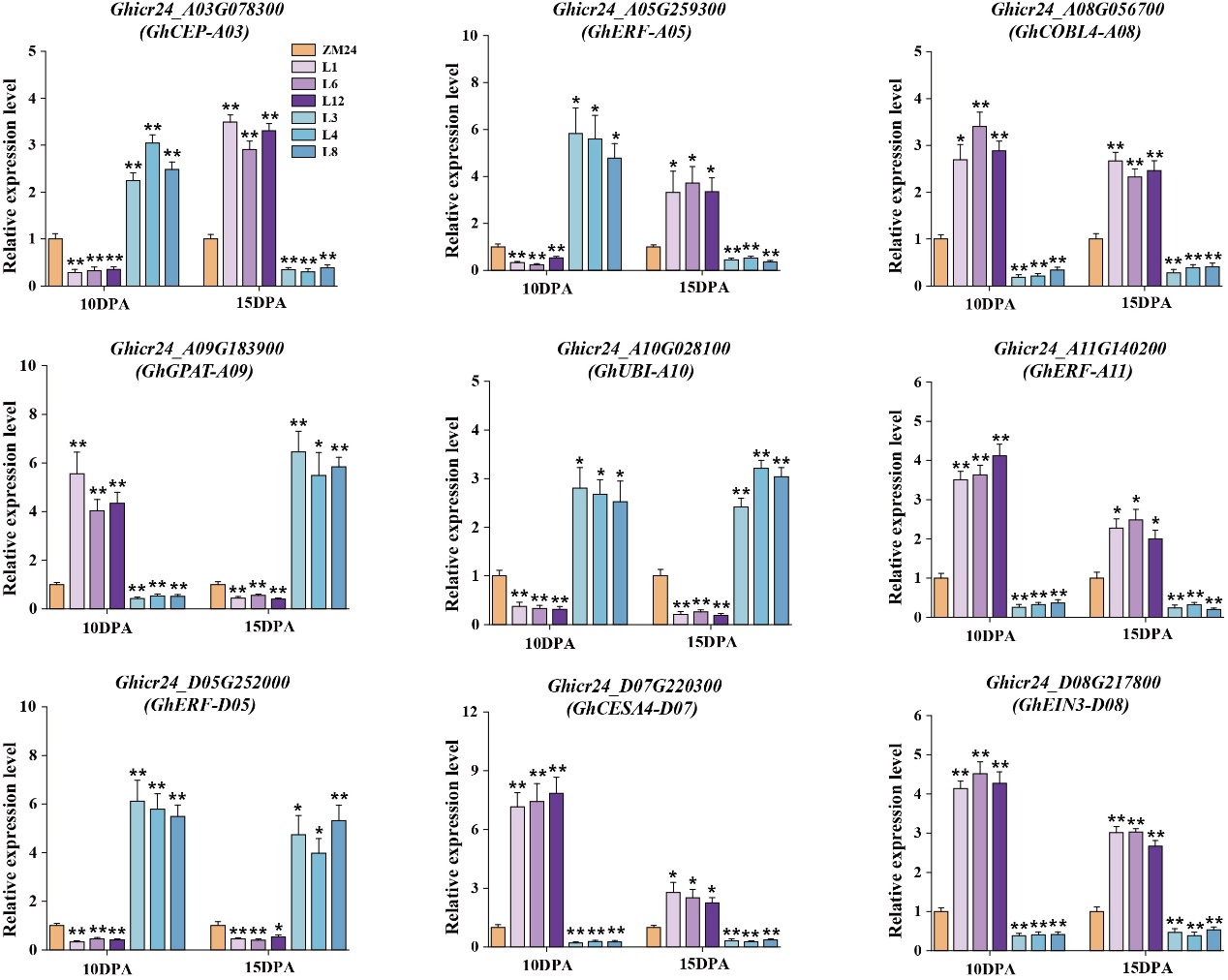


**Figures S16**. Expression levels of nine core ethylene-responsive genes in 10 and 15 DPA fibers from WT and ethylene overproduction (EO)/ethylene deficient (ED) lines. The presented data are the mean values and standard deviations from three independent biological replicates. Student’s *t*-tests were conducted and revealed significant differences (*, *P* < 0.05; **, *P* < 0.01) in gene expression levels between WT and EO lines as well as between WT and ED lines. ZM24 is the WT, while L1, L6 and L12 are EO lines and L3, L4 and L8 are ED lines


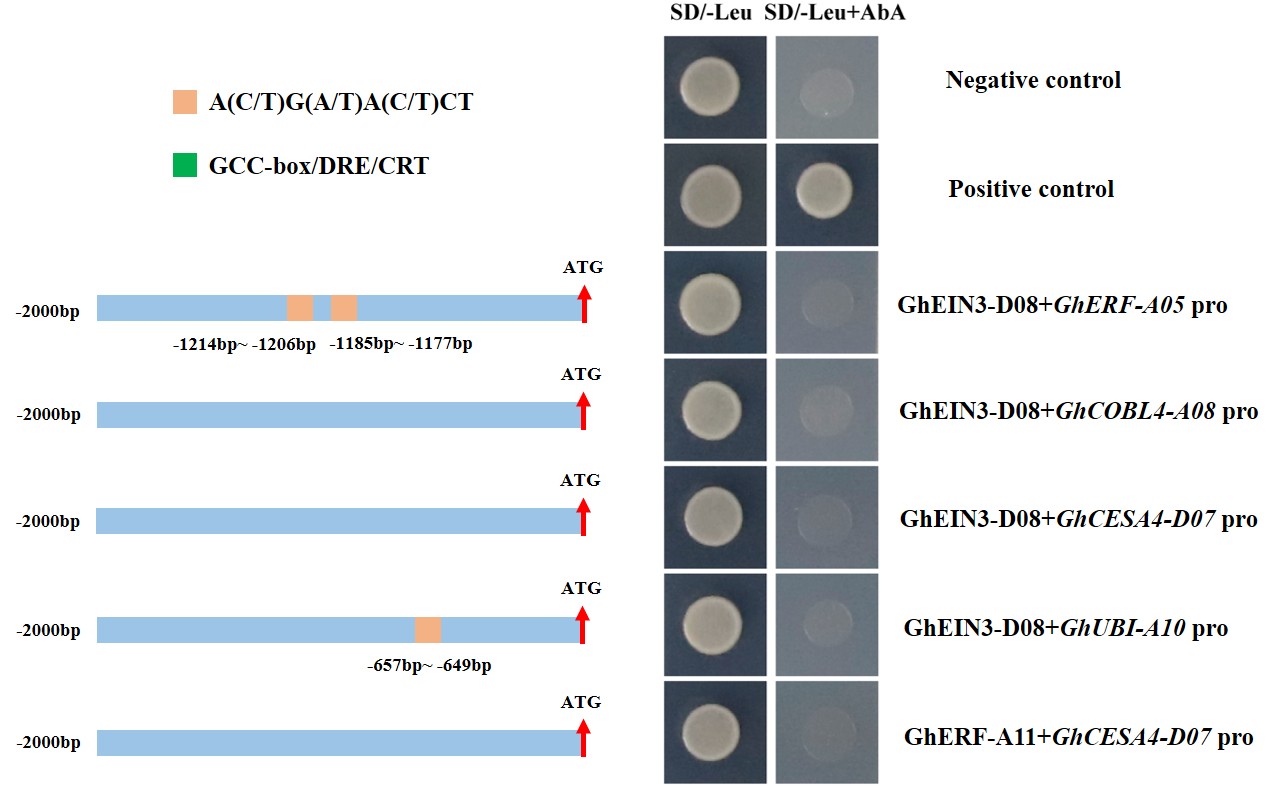


**Figures S17**. Yeast one-hybrid assay of ability of GhEIN3-D08 and GhERF-A11 to bind to the promoters of potential target genes and the distribution of EIN3- and ERF-binding elements in their promoters. Yeast transformants were selected on SD/-Leu medium and the higher stringency SD/-Leu with Aureobasidin A medium.


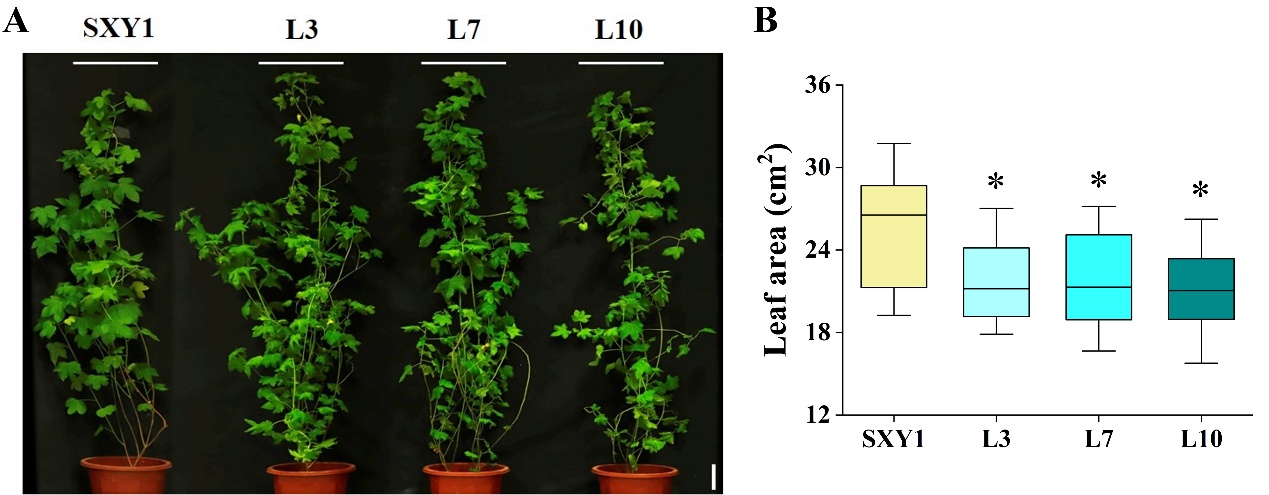


**Figures S18**. *PK1* overexpression in *G. arboreum* (SXY1) modestly reduces leaf blade area. (A) The SXY1 and *PK1* overexpression lines (*G.* *arboreum*) growing in soil. Bar = 10 cm. (B) Quantification of leaf blade area from fully expanded leaves collected at the same node position of SXY1 and *PK1* overexpression lines (n = 10); petioles were removed and laminae were imaged and measured in ImageJ. Student’s *t*-tests showed that the differences between the SXY1 and *PK1* overexpression lines were statistically significant (*, *P* < 0.05). SXY1 is the wild-type plant, while L3, L7 and L10 denote three independent lines of *PK1* overexpression transgenic cotton (*Gossypium arboreum*).


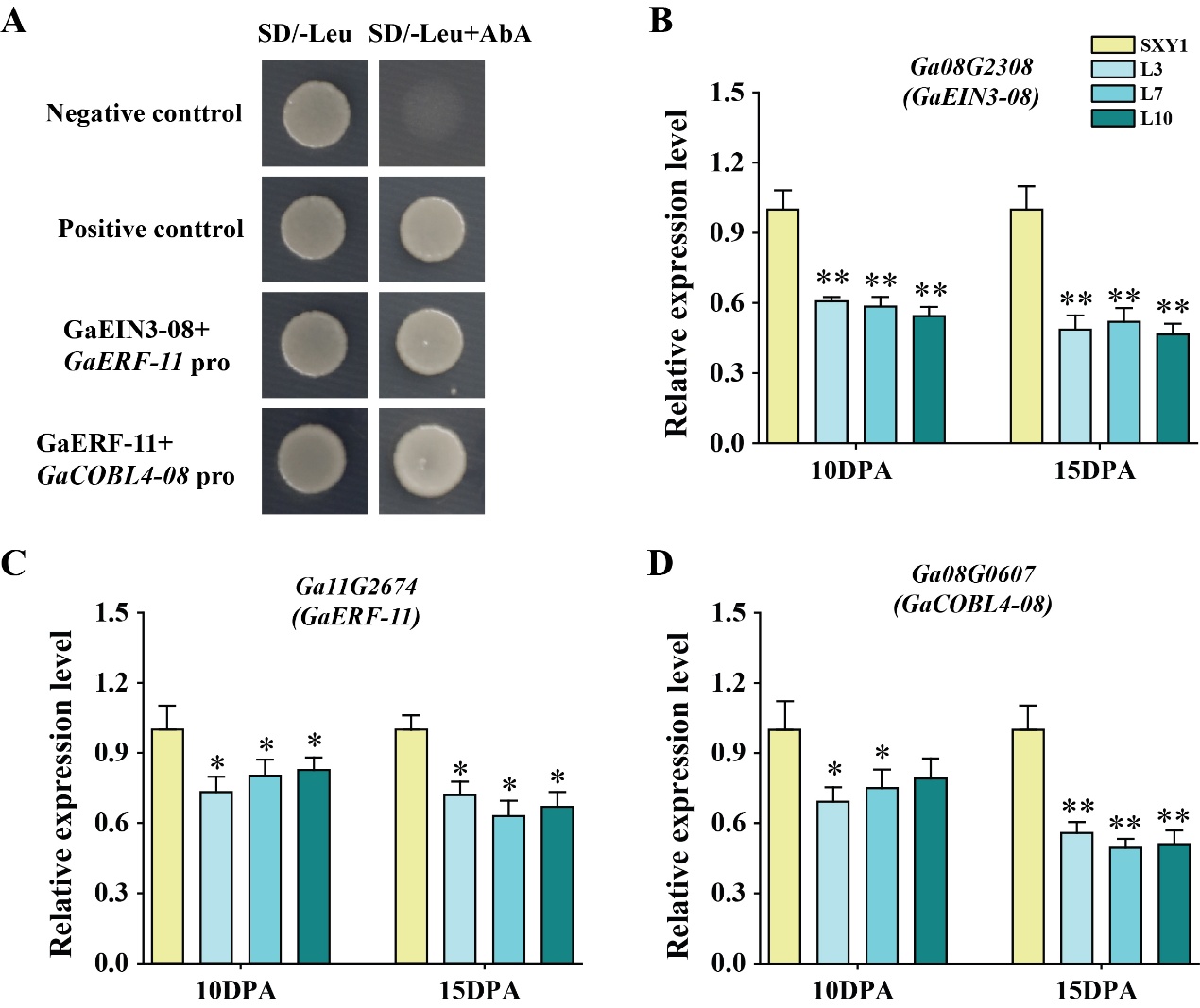


**Figures S19**. The EIN3-ERF-COBL4 cascade is conserved in *G. arboreum* but is down-regulated by elevated ethylene. (A) Yeast one‑hybrid assays. GaEIN3‑08 binds *GaERF‑11* promoter, and GaERF‑11 binds *GaCOBL4‑08* promoter. Yeast was grown on SD/‑Leu (control) or SD/‑Leu + AbA (selection). (B-D) Expression levels of *GaEIN3-08* (B), *GaERF-11* (C), and *GaCOBL4-08* (D) in 10 and 15 DPA fibers from SXY1 and *PK1* overexpression lines. The presented data are the mean values and standard deviations from three independent biological replicates. Student’s *t*-tests were conducted and revealed significant differences (*, *P* < 0.05; **, *P* < 0.01) in gene expression levels between SXY1 and *PK1* overexpression lines. SXY1 is the WT, while L3, L7 and L10 denote three independent lines of *PK1* overexpression cotton (*G. arboreum*)

**Table S6**. Comparison of fiber quality characteristics between ZM24 and *PK1* overexpression cotton.

| Year | Cultivar | Upper half mean length  [mm] | Fiber strength  [cN•tex-1] | Micronaire |
| --- | --- | --- | --- | --- |
| 2021 | ZM24 | 28.63 ± 0.28 | 28.12 ± 1.39 | 4.31 ± 0.17 |
|  | *PK1* OE | 26.61 ± 0.34^**^ | 30.32 ± 1.33^**^ | 4.48 ± 0.25 |
| 2022 | ZM24 | 29.24 ± 0.54 | 27.58 ± 1.27 | 4.49 ± 0.21 |
|  | *PK1* OE | 27.39 ± 0.76^**^ | 29.64 ± 1.38^**^ | 4.72 ± 0.18^*^ |
| 2023 | ZM24 | 28.89 ± 0.41 | 27.44 ± 1.25 | 4.53 ± 0.28 |
|  | *PK1* OE | 26.79 ± 0.45^**^ | 29.27 ± 1.68^*^ | 4.80 ± 0.33 |

The presented values represent the mean ± SD of ten independent replicates, with each replicate containing 10 g fiber. Student’s *t*-tests demonstrated that there were significant differences, ^*^ *P* < 0.05; ^**^ *P* < 0.01) between the ZM24 and *PK1* overexpression cotton.
